# RNA-binding protein NONO contributes to cancer cell growth and confers drug resistance as a theranostic target in TNBC

**DOI:** 10.7150/thno.45037

**Published:** 2020-07-02

**Authors:** Seong-Jin Kim, Jin-Sung Ju, Myoung-Hee Kang, Ji Eun Won, Young Ha Kim, Prahlad V Raninga, Kum Kum Khanna, Balázs Győrffy, Chan-Gi Pack, Hee-Dong Han, Hee Jin Lee, Gyungyub Gong, Yong Shin, Gordon B. Mills, Seong-il Eyun, Yun-Yong Park

**Affiliations:** 1 Department of Convergence Medicine, University of Ulsan College of Medicine, Seoul, Korea.; 2 Asan Institute for Life Sciences, Asan Medical Center, Seoul, Korea.; 3 Department of Immunology School of Medicine, Konkuk University, Chungju, South Korea.; 4 QIMR Berghofer Medical Research Institute, Brisbane, QLD, Australia.; 5 MTA TTK Lendület Cancer Biomarker Research Group; Institute of Enzymology; Semmelweis University 2nd Dept. of Pediatrics, Budapest, Hungary.; 6 Department of Pathology, University of Ulsan College of Medicine, Asan Medical Center, Seoul, Korea.; 7 Department of Cell, Development and Cancer Biology, Oregon Health & Science University, Portland, OR, USA.; 8 Department of Life Science, Chung-Ang University, Seoul, Korea.

**Keywords:** RBP, NONO, TNBC, STAT3, Auranofin.

## Abstract

Breast cancer (BC) is one of the most common cancers in women. TNBC (Triple-negative breast cancer) has limited treatment options and still lacks viable molecular targets, leading to poor outcomes. Recently, RNA-binding proteins (RBPs) have been shown to play crucial roles in human cancers, including BC, by modulating a number of oncogenic phenotypes. This suggests that RBPs represent potential molecular targets for BC therapy.

**Methods:** We employed genomic data to identify RBPs specifically expressed in TNBC. NONO was silenced in TNBC cell lines to examine cell growth, colony formation, invasion, and migration. Gene expression profiles in NONO-silenced cells were generated and analyzed. A high-throughput screening for NONO-targeted drugs was performed using an FDA-approved library.

**Results:** We found that the NONO RBP is highly expressed in TNBC and is associated with poor patient outcomes. NONO binds to STAT3 mRNA, increasing STAT3 mRNA levels in TNBC. Surprisingly, NONO directly interacts with STAT3 protein increasing its stability and transcriptional activity, thus contributing to its oncogenic function. Importantly, high-throughput drug screening revealed that auranofin is a potential NONO inhibitor and inhibits cell growth in TNBC.

**Conclusions:** NONO is an RBP upstream regulator of both STAT3 RNA and protein levels and function. It represents an important and clinically relevant promoter of growth and resistance of TNBCs. NONO is also therefore a potential therapeutic target in TNBC.

## Introduction

Breast cancer (BC) is the most common cancer in women worldwide 1, 2. The estrogen receptor (ER), progesterone receptor, and epidermal growth factor receptor, Her2, are key molecular markers and therapeutic targets in BC 1, 3. However, whereas ER-, PR- or Her2-positive BC patients can now be managed with targeted therapies, triple-negative breast cancer (TNBC) tumors, i.e., those that do not express any of these three key factors, lack molecular markers for diagnosis and therapeutic targets 4 for treatment. In addition, compared with hormone receptor-positive BCs, TNBCs show aggressive features that lead to rapid treatment failures 5. The only approved systemic treatment option for TNBC patients at present is chemotherapy 4. However, given the suboptimal treatment outcomes of these cases despite chemotherapy, targeted therapies for TNBC are urgently needed 6.

RNA-binding proteins (RBPs) have recently emerged as factors that control diverse molecular functions such as mRNA stability, splicing, translational efficiency, and protein subcellular localization, thus contributing to multiple cellular functions 7. Human genome data have revealed the existence of more than 1500 of these molecules, representing ~7.5% of all protein-coding genes 7. Although the mechanisms by which RBPs influence tumorigenesis have not yet been fully elucidated, they regulate multiple oncogenes and have thus been speculated to represent possible therapeutic targets for diverse human cancers including BC 8, 9. Our aim was to identify RBPs that exhibit selective functional roles in TNBC.

Based on gene expression profiles, we identified a novel TNBC-specific RBP, NONO, and here demonstrate that it exerts oncogenic effects by regulating STAT3 (signal transducer and activator transcription 3). Indeed, NONO was further found to stabilize STAT3 RNA and also modulate STAT3 transcriptional activity via its direct binding to the STAT3 protein. Furthermore, both NONO and STAT3 levels showed a negative association with chemotherapeutic drug responsiveness and the prognosis of TNBC patients. Our current findings provide important new mechanistic insights into the functional roles of NONO in TNBC through its regulation of STAT3 and suggest that this RBP could be a viable therapeutic target for overcoming treatment resistance in TNBC.

## Materials and Methods

### Cell lines

Human breast cancer cell lines used were obtained from the American Type Culture Collection (ATCC, Manassas, VA). Cells were maintained in Dulbecco's modified Eagle's medium (DMEM) or Roswell Park Memorial Institute (RPMI) 1640 medium (HyClone, Logan, UT) supplemented with 10% fetal bovine serum (FBS) and 1% antibiotic-antimycotic (Anti-Anti; Gibco, Gaithersburg, MD) in a humidified incubator containing 5% CO_2_ at 37 °C 10, 11.

### Cell proliferation assay

The indicated cell lines were seeded in triplicate in 96-well plates (3×10^3^ cells/well). Cell viability was measured using a cell counting kit (CCK-8 (CK04-20; Dojindo, Rockville, MD) following the manufacturer's recommended protocol. After the CCK-8 reagent was added, the cells were incubated at 37 °C for 1 h. The number of viable cells was assessed by measuring the absorbance at 450 nm.

### Colony forming assay

Cells were seeded in 6-well plates (1×10^3^ cells/well) and cultured in DMEM or RPMI media for 2~3 weeks. The cells were then fixed in methanol and stained with 0.05% crystal violet for 30 min to count cell colony numbers.

### Cell migration and invasion

For the cell migration and invasion assay, cells were seeded (4×10^4^ cells/well) on the upper chamber (#3422; Corning, Midland, NC) in media without FBS. The upper chambers were coated with Matrigel (#354234; Corning, Midland, NC) for the invasion assay since the cell migration assay does not include a coating step. The lower chamber contained the medium supplemented with 10% FBS. After incubation for 24 h, the cells on the underside were fixed with 4% paraformaldehyde and stained with 0.05% crystal violet. The Matrigel, the un-migrated, and the un-invaded cells were removed using cotton swabs.

### Wound healing assay

To measure cell migration using a wound healing assay, cells were seeded in 6-well plates (5×10^5^ cells/well) and incubated at 37 °C for 24 h. When the cells reached 100% confluence, the monolayers were scratched with sterile 200 μL tips and washed with medium to remove any detached cells. Images were captured at 0 and 12 h, and wound healing (%) was calculated and analyzed.

### Flow Cytometry Cell Cycle Analysis and EdU assay

Cells were trypsinized and collected by centrifugation. The cells were washed twice with PBS and fixed with cold 70% ethyl alcohol at -20 °C for 1 hour. After washed with PBS, the cells were treated RNase A, stained 20 µg/mL propidium iodide at 37°C for 30 min and analyzed with CytoFLEX (CytoFLEX; Beckman, Brea, CA,US) flow cytometer.

Click-iT^®^ EdU Alexa Fluor^®^ 488 Flow cytometry assay kit (C10632; Invitrogen Waltham, MA, US) was used to cell cycle analysis according to the manufacturer's protocol. EdU staining and flow cytometry cells were incubated with 10 µM EdU for 2 h. Harvested cells were fixed with 4% paraformaldehyde at RT for 15 min and permeabilized by saponin-based permeabilization buffer for 15 min. The cells were digested with a reaction solution containing Alexa Fluor^®^ 488 at was added at room temperature, in the dark, for 30 min and determined using CytoFLEX (CytoFLEX; Beckman, Brea, CA, US) flow cytometer.

### Sphere formation assay

Cells were seeded in ultra-low attachment 6-well plates (5×10^3^ cells/well) for 10 days. The spheres were cultured in DMEM/F12 medium, containing B27 supplement (Gibco, Gaithersburg, MD), 20 ng/mL epidermal growth factor, and 20 ng/mL-basic fibroblast growth factor. The spheres were then photographed and counted.

### Immunoprecipitation and western blotting

Immunoprecipitation (IP) and western blotting (WB) were performed as described previously 10-12. Briefly, cell extracts were prepared in lysis buffer (50 mM Tris at pH 7.4, 150 mM NaCl, 1% Triton ×100, and 1mM ethylenediaminetetraacetic acid [EDTA]) supplemented with protease inhibitors (Complete Mini, EDTA-free; Roche, Mannheim, Germany) and phosphatase inhibitors (Xpert Phosphatase Inhibitor Cocktail; GenDEPOT, Houston, TX, US). The lysates were then centrifuged at 13,000 rpm for 30 min at 4 °C, and supernatants were recovered. For IP, cell lysates were pre-cleared with protein A/G beads and then incubated for 4 h with protein A/G beads covalently coupled with NONO and anti-STAT3 antibodies. The immunocomplexes were washed four times with cell extraction buffer. Eluted samples or whole-cellular lysates were resolved by SDS-PAGE, and proteins were detected by WB using the indicated antibodies. The following antibodies were used in this study: NONO (Millipore 05-950; Burlington, MA or Bethyl #A300-582A [587A]; Montgomery, TX, US), STAT3 (Cell Signaling #12640; Danvers, MA, US, or Abcam #ab119352, Cambridge, UK), phospho-STAT3 (Cell Signaling #9131), β-actin (Cell Signaling #4967), FLAG (Cell Signaling #2368), and MYC (Cell Signaling #2276).

### Immunofluorescence microscopy

Immunofluorescence assays were performed as previously reported 12. The antibodies used for immunofluorescence staining were anti-NONO (#05-950, 1:500, Millipore) and anti-STAT3 (#12640, 1:500, from Cell Signaling).

### Short hairpin RNA (shRNA) transfection

ShNONO (TRCN0000286628; TRCN0000294049) and shGFP (SHC005) clones were purchased from Sigma (St. Louis, MO, US). 293FT cells were co-transfected with the lentiviral packaging plasmids psPAX2 and pMD2.G through the lentiviral vector using Lipofectamine® 2000 Reagent (Invitrogen). Viral supernatants were harvested 48 h after transfection and pooled. For infection of target cells, the filtered viral supernatant was diluted with culture medium supplemented with 8 μg/mL Polybrene. The infectious supernatant was removed after 24 h, and selection of infected cells was commenced after 48 h.

### Plasmids and luciferase assay

STAT3 cDNA, STAT3-reporter, and NONO cDNA have been described previously 13, 14. To construct the STAT3 3' UTR reporter plasmids, oligonucleotides covering the putative NONO binding site were designed as described in Figure 4A and S6 as follows: M1-1 wild-type forward; 5'- AAACTAGCGGCCGCTAGTCTGTCTCCAGGCAGGAGGACTT-3', reverse: 5'-CTAGAAGTCCTCCTGCCTGGAGACAGACTAGCGGCCGCTAGTTT-3'; M1-2 wild-type forward: 5'-AAACTAGCGGCCGCTAGTCTACCTTCAGGCAGGTCCTACT-3', reverse: 5'-CTAGAGTAGGACCTGCCTGAAGGTAGACTAGCGGCCGCTAGTTT-3'; M2 wild-type forward: 5'-AAACTAGCGGCCGCTAGTCTCTGCTCCTGGAACACACCTT-3'; reverse: 5'-CTAGA AGGTGTGTTCCAGGAGCAGAG ACTA GCGGCCGCTAGTTT-3'; M3 wild-type forward: 5'-AAACTAGCGGCCGCTAGTGAACCTGGGAGGCGGAGGTTGT-3', reverse: 5'-CTAGACAACCTCCGCCTCCCAGGTTCACTA GCGGCCGCTAGTTT-3'; M4 wild-type forward: 5'-AAACTAGCGGCCGCTAGTGTAATCCCAGCACTGTGGGAGT-3', reverse: 5'-CTAGA CTCCCACAGTGCTGGGATTACACTAGCGGCCGCTAGTTT-3'. Oligonucleotides containing the restriction sites for *PmeI* and *XbaI* were annealed and cloned into pmirGLO Dual-Luciferase Expression Vectors (#E1330; Promega, Madison, WI, US). To introduce point mutations as depicted in Figure 4A in the seed region of the NONO binding site, mutant oligos were cloned into pmirGLO vectors. The sequences were verified using an automatic sequencer. For luciferase-based reporter assays, cells were transfected with reporter genes and plasmids using the Dual-Glo® Luciferase Assay System (E2940; Promega) and Dual-Luciferase® Reporter Assay System (E1910; Promega) in accordance with the manufacturer's instructions. After 48 h, the cells were harvested to measure luciferase activity, which was normalized to that of *Renilla* (*n*=3).

### Microarray

Microarray analysis was performed as described previously 10-12. Briefly, total RNA was isolated from the indicated cell lines using a mirVana RNA isolation kit (Ambion, Inc. Austin, TX, US). Labeling and hybridization were executed on 500 ng of total RNA, in accordance with the manufacturer's protocols (#AMIL1791, Ambion, Inc.). Labeled RNA was hybridized with bead chips, which were then washed and scanned with an Illumina BeadArray Reader (Illumina, Inc. Sam Diego, CA, US). The microarray data were normalized using the quantile normalization method in the Linear Models for Microarray Data (LIMMA) package in the R language environment. The expression level of each gene was transformed into a log_2_ base before further analysis, and the data were deposited in Gene Expression Omnibus (GEO, GSE117927).

### Quantitative real-time reverse transcription polymerase chain reaction (qRT-PCR)

RNA was isolated by Trizol extraction in accordance with the manufacturer's instructions (Invitrogen). Quantitative PCR was performed with gene-specific TaqMan primers using an ABI prism StepOne^TM^ Real-Time PCR system and the SensiFAST^™^ Probe Hi-ROX One-Step Kit (Bioline; London, UK) for gene expression analysis. Each value was normalized to the human peptidylprolyl isomerase A gene expression. The following primers were used in this study: PPIA (ABI, Hs0419421-S1; Foster City, CA), NONO (IDT, Hs, PT.58.25447000; Skokie, IL), STAT3 (IDT, Hs, PT.58.3750282), CCNB1 (ABI, Hs0103099_m1), CCND1 (ABI, Hs00765553_m1), NANOG (ABI, Hs04399610_m1), and OCT4 (Hs00742896_m1).

### Statistical analysis of microarray data and survival analysis

The Class Comparison method in the BRB-Array Tools package was used to identify genes differentially expressed between two array groups. Differences in gene expression in the profile data were considered statistically significant if the *p*-value was less than 0.001. Clustering analysis was performed with Cluster and visualized with TreeView. Kaplan-Meier plots were used to estimate correlations with patient survival, while the cutoff standard of gene expression value for survival analysis was estimated based on the best cutoff value with R package 15.

### Chromatin immunoprecipitation

Chromatin immunoprecipitation assays were performed using a Magnetic ChIP assay kit (Pierce #26157; Waltham; MA, US) in accordance with the manufacturer's instructions. MDA-MB-231 cells were cross linked with 1% formaldehyde for 10 min and quenched using glycine. The cells were then washed with cold PBS and treated with 1.5μL of micrococcal nuclease at 37 °C for 15 min. The protein-crosslinked chromatin was immunoprecipitated with the indicated antibodies and the retrieved DNA was then analyzed by qPCR using the following primers for the *CCND1* promoter: forward 5'- CGAACACCTATCGATTTTGCTAA-3' reverse, 5'-TTGACCAGTCGGTCCTTGCGG-3'.

### RNA interference by siRNA

The target sequences in the siRNA directed against NONO and in a non-specific siRNA were as follows: siNONO-1: 5′-CUCAGUAUGUGUCCAACGA-3′; siNONO-2: 5′-CAAACGUCGCCGAUACUAA-3′; si NONO-3: 5′-GAUGGAAGCUGCACGCCAU-3′; siCon: 5' UUCUCCGAACGUGUCACGU-3'. The cells were transfected with 100 pmol of siRNA (Sigma, St. Louis, MO, US) for 48 h using Lipofectamine® RNAiMAX Reagent (Invitrogen) in accordance with the manufacturer's instructions.

### RNA-immunoprecipitation (RNA-IP)

Cells were cultured to ~ 80-90% confluency in 15-cm plates and washed with PBS. RNA-IP was performed using a Magnetic Chromatin Immunoprecipitation kit (#53024) from Active Motif (Carlsberg, CA, US) in accordance with the manufacturer's protocol. The antibodies used were anti-rabbit-NONO and anti-rabbit-IgG. Immunoprecipitated RNA was purified using EZBlue (Sigma-Aldrich, St. Louis, MO, US) and treated with DNase1. The immunoprecipitated RNA was quantified (qPCR kit) with a STAT3 probe (IDT, Hs, PT.58.3750282).

### Preparation of the CH-NP (Chitosan-nanoparticle)

Chitosan (CH, low molecular weight; deacetylation degree, 75-85%), sodium tripolyphosphate (TPP), and acetic acid were purchased from Sigma-Aldrich (St. Louis, MO). Preparation of the siRNA-incorporated CH-NP depended on the electronic interaction between cationic CH, anionic TPPa and siRNA. Briefly, predetermined concentrations of TPP (0.25% w/v) and siRNA (1 μg/μL) were added to CH (2 mg/mL, 1% acetic acid) solution, and the CH-NP/siRNA formed spontaneously as the mixture was stirred continuously at 25 °C. After incubation at 4 °C for 30 min, the CH-NP/siRNA was collected by centrifugation (Hanil Science Industrial, Seoul, Korea) at 13,000 rpm for 50 min at 4 °C. The size and surface charge of CH-NP were measured by dynamic light scattering using an electrophoretic light scattering photometer (SZ-100, HORIBA, Kyoto, Japan). Encapsulation efficiency of siRNA was measured with an ND-1000 spectrophotometer (NanoDrop Technology, Wilmington, DE) at 260 nm using the supernatant after centrifugation of CH-NP.

### Antitumor efficacy of CH-NP-NONO siRNA

Female BALB/c nude mice (7 weeks old, 20 g) were purchased from OrientBio (Gapyeong, South Korea). All animal procedures and maintenance conditions were approved by the Konkuk University Institutional Animal Care and Use Committee (#KU17188). To induce tumors, MDA-MB-231 (1 × 10^6^ cells/50 µL HBSS) cells were injected subcutaneously into the mice (*n*=5 mice per group). CH-NP-control siRNA (5'-UUCUCCGAACGUGUCACGU[dT][dT]-3') or CH-NP-NONO siRNA (5'-GAUGGAAGCUGCACGCCAU[dT][dT]-3') was administered twice weekly via intravenous injection at a dose of 5 μg of siRNA per mouse. Treatments continued until the control group became moribund (typically 4 to 5 weeks), at which point all the mice were sacrificed. The tumor volume was measured using calipers and calculated with the formula: tumor volume (mm^3^) = length × (width)^2^/2; the tumor weights of the tumors were also recorded.

### Protein structural homology modeling

Homology-based structural modeling of NONO (accession ID: NP_001138880.1) and STAT3 (accession ID: NP_001356441) was performed using the SWISS-MODEL web server (http://swissmodel.expasy.org) 16. Human SFPQ (PDB ID: 4WIJ) and mouse STAT3 (PDB ID: 1BG1), were selected as templates for NONO and STAT3 (the sequence similarities are 73.6% and 99.8%). The QMEAN4 Z-scores given by SWISS-MODEL were 1.61 and -2.41. Computational docking simulations were conducted with ClusPro 2.0 using the hydrophobic-favored scoring scheme 17. Molecular docking analyses were performed using AutoDock Vina (ver. 1.1.2) which is one of the most widely used methods for protein-ligand docking. The file format was determined using AutoDock Tools (ver. 1.5.6) (Scripps Research Institute CA, USA). Binding affinities for those compounds were evaluated using the negative Gibbs free energy (ΔG) scores (kcal/mol)^18^. Graphical representations of the docking structures were constructed using PyMOL (ver. 1.3; DeLanoScientific, San Carlos, CA, US).

### High throughput drug screening

The pCDH-GFP-NONO-viral vector was used to infect MDA-MB-231 cells, which were seeded at a density of 1×10^3^ cells/well in a 96-well plate. After 24 h of incubation, the cells were exposed to the drug library compounds (BML-2843-0100; Enzo) at a final concentration of 5 µM in 0.5% dimethyl sulfoxide (DMSO) (v/v) (Sigma-Aldrich, St Louis, MO). After a 24 h incubation, the cells were fixed with 4% paraformaldehyde (PFA; Sigma-Aldrich) and washed with Dulbecco's phosphate-buffered saline (DPBS; Welgene; Korea) for 10 min. GFP intensities were determined with the Operetta High Content Screening System (Perkin Elmer, Waltham, MA) and analyzed by Harmony 3.5.1 high content imaging and analysis software (Perkin Elmer).

### Chemicals

Auranofin (#A6733), digoxin (#D6003), colchicine (#C9754), and podophyllotoxin (#P4405) were purchased from Sigma (St. Louis, MO, US).

### Caspase 3/7 activity assay

The indicated cell lines were seeded in white 96well plates (4×10^3^ cells/well). Cell caspase 3/7 activity was measured with the Caspase-Glo ® 3/7 activity assay (G8091; Promega, Madison, WI, US) according to the manufacturer's manual. After reagent was added, the cells were incubated at room temperature for 30 min. Luminescence intensity was measured using an Alpha PLUS(Multi-Label) Plate Reader (EnSpire, Pekin Elmer. Waltham, MA, US).

### Tissue microarray (TMA) and immunohistochemistry (IHC)

TMA and IHC were performed as previously described 9, 19 for patients who underwent surgery for primary BC between 1993 and 1998 at Asan Medical Center, Seoul, Korea. NONO antibody was used for TMA (1:200 dilution; Bethyl #A300-582A; Montgomery, TX, US).

## Results

### Identification of NONO as an oncogenic RBP in TNBC

**Figure 1:**
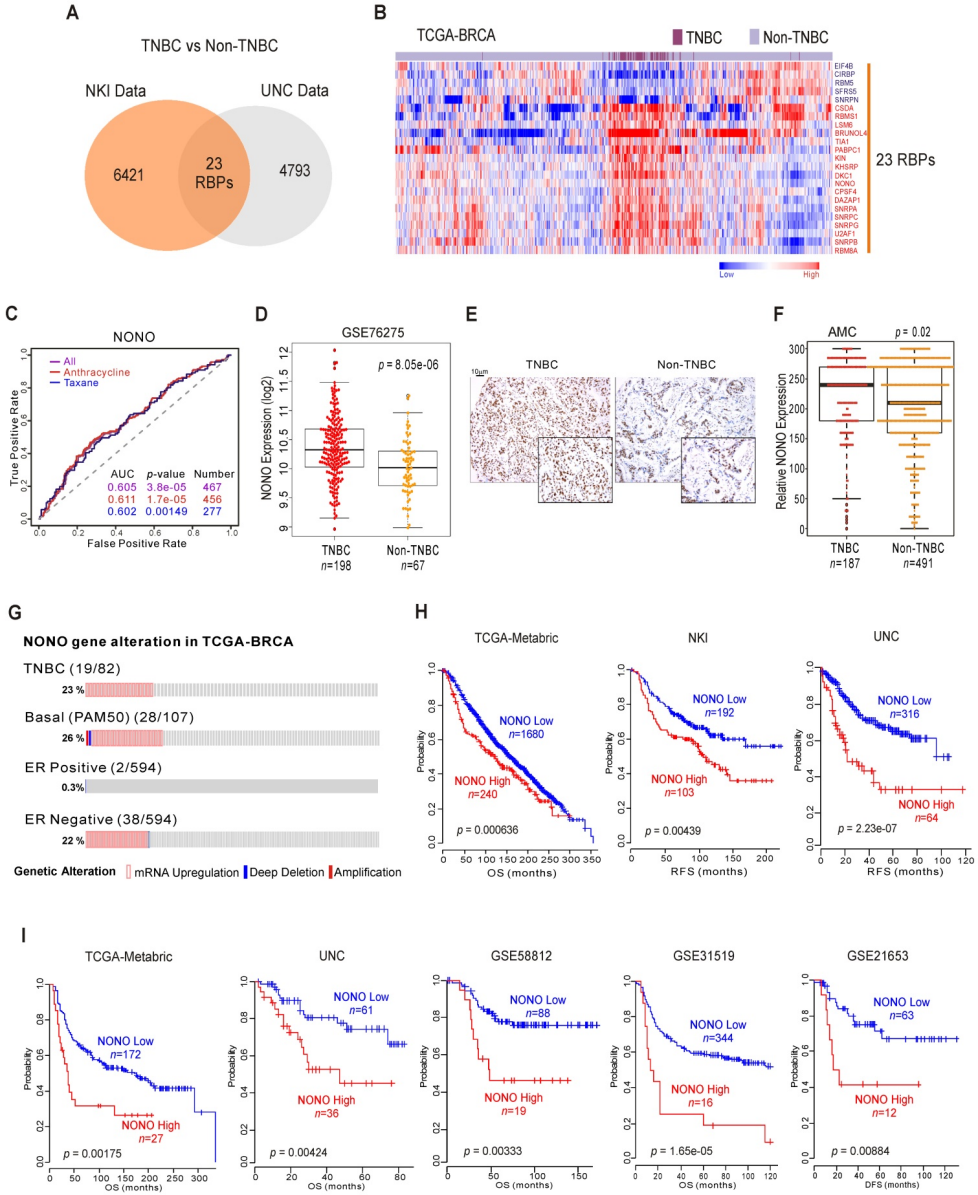
** NONO expression and prognosis in TNBC (A)** Venn diagram of genes showing significant differential expression between TNBC and non-TNBC in two independent BC patient cohorts. A univariate test using Class Comparison Analysis in the BRB array tool was employed. **(B)** The expression of 23 RBP genes was commonly up- or downregulated in the TCGA-BRCA cohort. Heat maps represent RBP expression levels. RBP genes are highlighted in blue or red text. **(C)** ROC curve analysis of NONO expression-related probability of recurrence in the BC cohorts. ROC curve analysis was performed to evaluate the correlation of NONO gene expression levels with chemo-response recurrence by determining the area under curve estimated through the concordance index. The corresponding *p-*values were determined using a one-sided Wilcoxon's rank test. **(D)** NONO mRNA expression (Log_2_) levels in TNBC and non-TNBC patients.** (E)** Representative image of NONO from TMA** (F)** Quantitative analysis of TMA data for NONO from breast tissues. Relative expression is multiply of stained intensity and percentage. **(G)** Gene alteration of NONO in the TCGA-BRCA cohort. **(H and I)** Patients in the indicated BC cohorts including TNBC cohorts (H) or TNBC-specific cohorts (I) were divided in two groups by relatively high or relatively low NONO expression and were considered for plotting. Statistically differences between these groups were indicated with log‐rank test.

RBPs have emerged as potential therapeutic targets in TNBC because of their proposed oncogenic functions 20. We screened human genomic data for TNBC-specific RBPs by applying approaches tested previously to uncover unknown functions of cancer genes 10, 11. TNBC has poorer prognosis compared to other types of BC (Figure S1A), and we wished to identify RBPs that are differentially expressed between TNBC and other BC types using the previously reported NKI 21 and UNC 22 cohorts. We compared the two BC subtypes (TNBC vs non-TNBC) using Class Comparison Analysis 23. We thereby identified 23 RBPs as potentially TNBC-associated (Figure 1A and Figure S1B). These results were validated in a cohort from The Cancer Genome Atlas Breast Invasive Carcinoma (TCGA-BRCA) (Figure 1B), suggesting that individual RBPs are BC subtype-specific. Using ROC (receiver operating characteristic) curves 24, we selected from our panel of candidates the RBPs that were significantly associated with chemotherapy-responsiveness, an important indicator of patient survival in TNBC. Ten RBPs were found to be associated with chemo-responsiveness (Figure S1C), among which NONO showed the best performance in terms of predicting TNBC, as it had the lowest *p*-value (Figure 1C).

NONO/nrb54 is a non-POU domain-containing octamer-binding protein that has diverse molecular functions in gastric 25, prostate 26, and lung cancer 27. However, the mechanisms underlying its functional roles in cancer and specifically in TNBC have not been fully identified. As shown in Figure 1D and Figure S2A, independent BC datasets indicate that the NONO mRNA levels are significantly upregulated in TNBC compared with other BC types; these data were also validated by TMA results (Figure 1E-F). In addition, our analysis also revealed that NONO expression is higher in tumor tissues compared with normal tissues (Figure S2B) as previously reported 28.

Next, we examined the NONO expression profile in the TCGA-BRCA cohort. As expected, NONO expression was higher in ER-negative patients, including in TNBC cases (Figure 1G). We also detected NONO mRNA up-regulation and amplification in 23% of the TNBC samples and in 26% of the basal-like subtype specimens. However, increased NONO levels were notable observed in only 0.3% of the ER-positive BC cases. We next investigated whether NONO is associated with patient survival in BC, including TNBC. Low NONO levels showed an association with improved survival in the whole cohort (Figure 1H and Figure S3A) 29, likely due to the poorer survival outcomes of patients with TNBC, in which NONO is highly expressed. Importantly, among the TNBC cases, a lower NONO level was associated with improved outcomes across multiple TNBC datasets (Figure 1I and Figure S3B). In addition, TCGA data analysis revealed that NONO prognosis was more specific in TNBC compared with non-TNBC patients (Figure S3C), and multivariate analysis with multiple clinical parameters revealed that NONO expression is significantly correlated with patient prognosis (Figure S3D). Taken together, the data indicate that NONO expression is strongly negatively correlated with a favorable prognosis in TNBC and may be a good indicator of clinical outcomes in patients with these tumors.

### NONO influences TNBC cell growth

**Figure 2:**
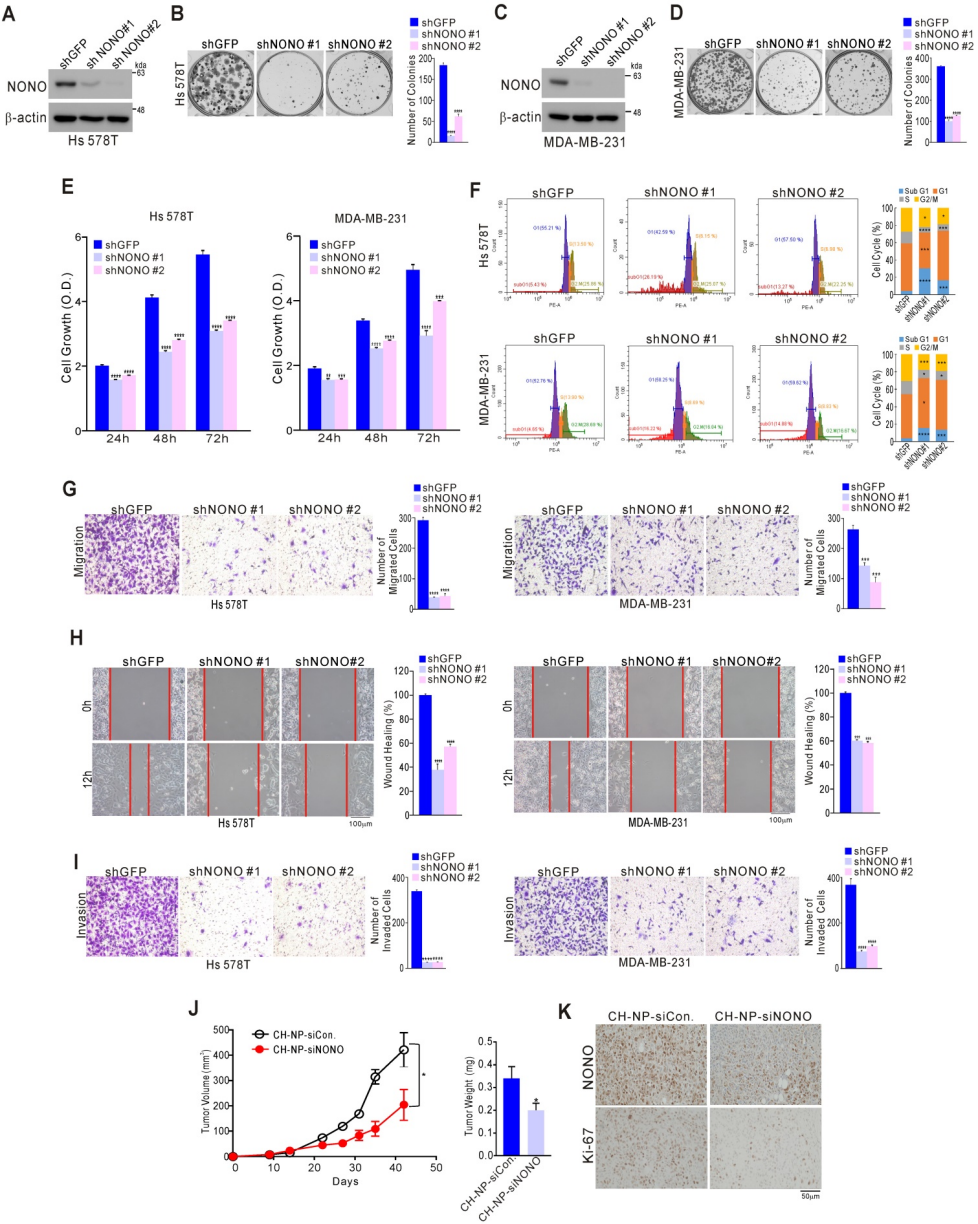
** NONO regulates cell growth and motility in TNBC cells (A-D)** The indicated TNBC (triple-negative breast cancer) cells were stably transfected with shNONO or control shRNA (shGFP) and then analyzed by western blotting with a NONO antibody **(A, C)**. The clonogenic survival of infected cells was quantified by colony formation assay **(B, D)**. **(E)** Infected cells were analyzed by proliferation assay (CCK8 assay) and FACS** (F)**. **(G)** Infected cells were tested in cell migration assays using Boyden chambers. These were conducted without extracellular matrix for 24 h and the migratory capacity of the cells was quantified by the number of stained cells. **(H)** The migration of infected cells was analyzed via a wound healing assay performed for 0 and 12 h by measuring the areas without cells. **(I)** Cell invasion was analyzed using Boyden chambers, with Matrigel functioning as the extracellular matrix. The cells in the invasion assay were incubated for 24 h at 37 ^o^C and stained with crystal violet, following which, they were quantified. **(J)** After MDA-MB-231 cells were injected into nude mice, the indicated siRNA was administered to the animals. Tumor volumes and weights were then measured (*n*=5). **(K)** Representative immunohistochemistry analysis of mouse samples was performed. All results are shown as means plus standard deviations from three independent replicates (*p < 0.05, **p < 0.01, ***p < 0.005, and ****p < 0.001).

We examined the impact of NONO on TNBC cell growth to investigate whether it possesses oncogenic functions. A series of TNBC cell lines including Hs 578T, MDA-MB-231, and MDA-MB-468 were stably infected with shNONO lentivirus vectors to silence NONO expression (Figure 2A, 2C, and S4A). This led to significant inhibition of colony formation and cancer cell growth for each of these cell lines (Figure 2B, 2D, 2E, and S4B-C). Similar results were obtained using different siRNA molecules targeting NONO (Figure S5A-D). Genes with oncogenic potential frequently alter the cell cycle distribution. Indeed, NONO-silenced cells showed marked enhancement of the sub-G1 fraction consistent with induction of apoptosis, as well as pronounced reduction in the cells in S-phase (Figure 2F and S5E); caspase activity was also increased in NONO-silenced cells compared with parental cells (Figure S5F), indicating that NONO influences cancer cell growth by modulating apoptotic activity. Oncogenic potential is also frequently associated with cancer cell migration and invasion. Strikingly, NONO silencing significantly inhibited cell migration in transwell and wound healing scratch assays (Figure 2G and H). In addition, a cell invasion assay using Matrigel-coated transwells revealed that NONO silencing significantly suppressed TNBC cell invasion (Figure 2I). We also examined whether NONO influences tumor growth: in a mouse model, siRNA nanoparticles (CH-NP; Chitosan Nanoparticles)-led NONO knockdown led to tumor growth inhibition *in vivo* (Figure 2J) and to downregulation of the cell proliferation indicator Ki-67 (Figure 2K). Taken together, these results demonstrated that NONO has oncogenic properties as it can modulate the growth, migration, and invasion of TNBC cells, suggesting that suppression of this RBP might be an effective way to inhibit cancer cell growth in this aggressive type of BC.

### NONO regulates STAT3 expression in TNBC

**Figure 3:**
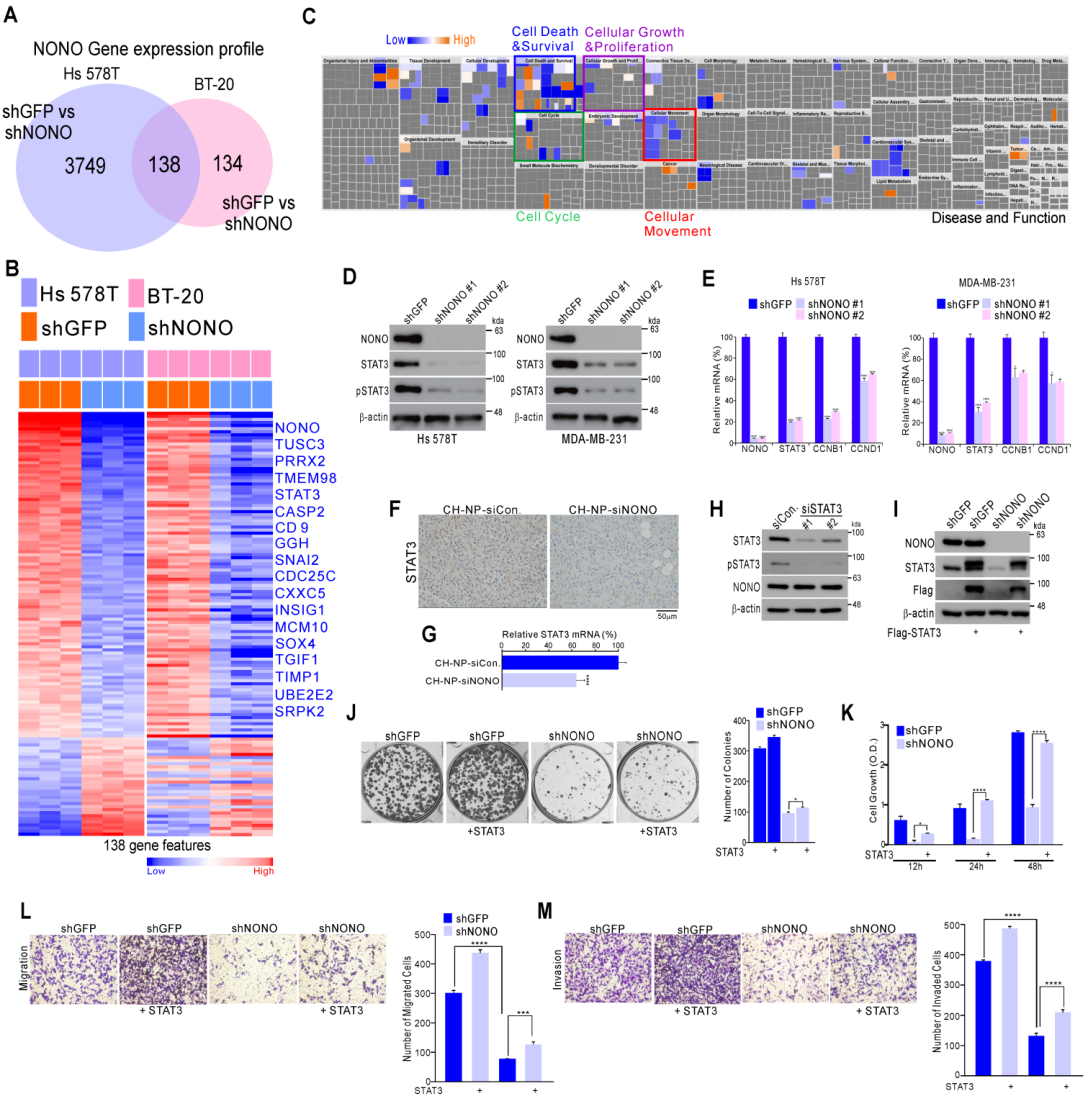
** NONO regulates STAT3 gene expression and thereby governs TNBC cell growth (A)** Gene expression signatures specific to the loss of NONO expression via shNONO in two TNBC cell lines. Genes in the Venn diagram were selected by applying Class Comparison Analysis from the BRB array tool (p < 0.001). **(B)** Gene expression profile presented in a matrix format. In this matrix, red and blue indicated relatively high and low expression, respectively, as indicated in the scale bar (log_2_-transformed scale). Genes with oncogenic potential are listed. **(C)** Ingenuity Pathway Analysis (IPA) of the genes differentially expressed after NONO silencing. **(D-E)** Western blot and** (D)** qRT-PCR** (E)** analysis of STAT3-associated genes in TNBC cells after infection with the indicated lentivirus.** (F-G)** Representative immunohistochemistry image **(F)** and qRT-PCR **(G)** analysis after silencing of NONO in a xenograft model.** (H)** Western blot analysis of NONO and STAT3 after treatment of MDA-MB-231 cells with siSTAT3. (I-M) Rescue experiments following the introduction of STAT3. After infection with shGFP or shNONO, the Flag-STAT3 plasmid was transfected into MDA-MB-231 cells. This was followed by western blotting (I), CFA (J), CCK8 (K), migration (L) and invasion (M) assays. Data represent the means plus standard deviations from three independent replicates. A student's t-test was used to examine statistical significance (*p < 0.05, **p < 0.01, ***p < 0.005, and ****p < 0.001).

To further investigate the mechanisms underlying the effects of NONO on cancer cell growth, we generated gene expression profiles in NONO-silenced Hs 578T and BT-20 cells. This profiling identified 138 mRNAs that are differentially expressed in response to NONO silencing in both TNBC cell lines (Figure 3A and Table S1). These transcripts included a number of previously identified oncogenic factors such as* TUSC3, PRRX2, STAT3, SOX4*, *TGFI1*, and others (Figure 3B). Ingenuity Pathway Analysis (IPA) indicated that the 138 NONO-regulated genes were functionally associated with cell growth and proliferation, cell cycle, and cell movement and migration, consistent with our findings shown in Figure 2 (Figure 3C). Among the oncogenic factors detected in these experiments, we focused on STAT3 as a downstream target of NONO, since it is a well-known oncogenic transcription factor (TF) related to TNBC functions 30, 31 and is an attractive therapeutic target currently being explored in multiple trials 32, 33. We investigated the role of NONO in regulating STAT3 signaling using western blotting and qRT-PCR to validate the gene expression profiling data. This analysis revealed that STAT3 itself, and its targets such as *CCNB1* and *CCND1*, were significantly downregulated in Hs 578T and MDA-MB-231 TNBC cells following shNONO infection (Figure 3D and 3E). In addition, STAT3 expression decreased after *in vivo* NONO silencing (Figure 3F and 3G). However, STAT3 did not influence NONO expression (Figure 3H), suggesting that NONO is an upstream regulator of STAT3.

We next evaluated whether the oncogenic properties of NONO were due to the activation of STAT3. Whereas TNBC cell growth, invasion, and migration was diminished by NONO silencing, re-introduction of the STAT3 gene reversed this effect (Figure 3I-3M), indicating that NONO-induced growth is dependent on STAT3 expression. Taken together, our current data suggest that NONO positively regulates STAT3 signaling and that this contributes to the oncogenic properties of NONO.

### Mechanism of STAT3 regulation by NONO in TNBC

**Figure 4:**
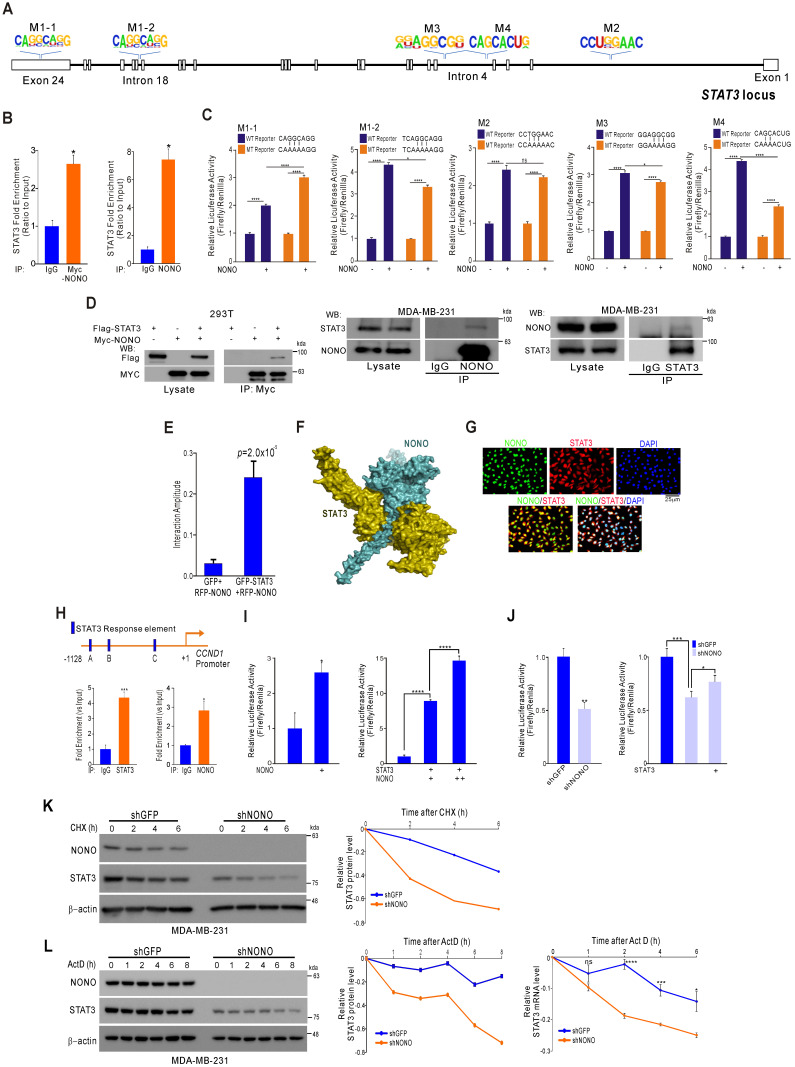
** NONO directly mediates STAT3 function in TNBC cells (A)** Alignment of the STAT3 locus sequence. **(B)** RNA-IP was performed with anti-Myc ab or endogenous NONO ab in Myc-NONO-overexpressing MDA-MB-231 cells or MDA-MB-231 cells. After RNA-IP, the cells were analyzed by qRT-PCR with the indicated probes. **(C)** A dual-luciferase assay in HEK293T cells, which harbored a luciferase reporter vector containing the wild or mutant-type sequence of STAT3 locus. Luciferase activities were measured after transfecting the indicated constructs. **(D)** HEK293T cells were transfected with Flag-STAT3 or Myc-NONO alone or in combination. The cells were then lysed and co-immunoprecipitated with Myc ab, and western blotting was performed with Myc and Flag antibodies (left panel). MDA-MB-231 cell lysates were immunoprecipitated with IgG and NONO (center panel) and STAT3 (right panel) antibodies, and western blotting was performed with STAT3 and NONO antibodies. **(E)** Protein interaction amplitudes based on the correlation functions obtained in the cells co-expressing GFP and RFP** (F)** Computational docking model for human NONO (cyan) and STAT3 (olive) predicted using ClusPro 17 (see Materials and Methods). **(G)** Cell localization of NONO and STAT3 in MDA-MB-231 cells. The cells were immunostained with the indicated antibodies and visualized using microscopy. **(H)** Schematic of the CCND1 promoter region. ChIP assays were performed in MDA-MB-231 cells using a STAT3 or NONO antibody. Recruitment of NONO to the CCDN1 promoter via STAT3 was analyzed using primers specific to this promoter. IgG was used as an internal control. **(I)** Dual‐luciferase reporter gene assay to determine the STAT3 activity level following transfection of NONO, STAT3, and a STAT3-reporter into HEK293T cells. **(J)** The STAT3-reporter was transfected into shNONO (or shGFP) infected MDA-MB-231 cells and rescued by STAT3 re-introduction. The cells were then used to measure luciferase activity. **(K and L)** MDA-MB-231 cells were stably transfected with shNONO or shGFP. After transfection, the cells were treated with DMSO, cycloheximide (CHX; 50μg/ml), or actinomycin D (Act D; 1μM) and harvested at the indicated time points. Total proteins or RNAs were extracted from the indicated cells and analyzed by western blotting with the indicated antibodies or qRT-PCR, respectively. Western blot bands were quantified using the software ImageJ. STAT3 protein levels were normalized to those of β-actin. The relative STAT3 protein or RNA level was designated as the protein or RNA half-life. All results are shown as means plus standard deviations from three-independent replicates (*p < 0.05, **p < 0.01, ***p < 0.005, and ****p < 0.001).

Although STAT3 was confirmed to be regulated by NONO in TNBC (Figure 3), we sought to understand more clearly the mechanism by which NONO regulates STAT3 gene expression to maintain its oncogenic functions in TNBC. RBPs usually bind directly to the 3' UTR region of their downstream target gene RNAs and previous reports have demonstrated that NONO binds to a specific response element within the locus 34. Based on this defined sequence, we explored NONO binding sites at the STAT3 locus, which we then ranked based on the degree of consensus (Figure S6). We identified five putative target sites (Figure 4A) and then demonstrated using RNA-IP that NONO binds STAT3 RNA in TNBC cells (Figure 4B). To further explore this interaction, we generated reporter constructs harboring the STAT3 locus containing NONO-binding sites. Wild type and mutant reporter constructs were generated and co-transfected with NONO cDNA into the cells. NONO significantly enhanced the activity of the wild type reporter. Of the NONO-binding sites that were mutation-tested, the M4 site (*CAGCACUG*) mutant showed the least reporter activity, suggesting that it is crucial for NONO binding to STAT3 RNA (Figure 4C).

STAT3 interacts with diverse proteins to maintain physiological functions in cancer cells as a TF 35. We investigated whether NONO regulates STAT3 function in addition to the RNA levels. Surprisingly, following endogenous and exogenous expression, STAT3 and NONO proteins were found to coimmunoprecipitate (Figure 4D), indicating a direct protein-protein interaction. To quantify this interaction more precisely, we performed fluorescence cross-correlation spectroscopy (FCCS) analysis in live cells that co-expressed GFP-STAT3 and RFP-NONO. The strength of the interaction is represented in this experiment by the relative cross-correlation amplitude. A significant interaction was detected between GFP-STAT3 and RFP-NONO compared with the corresponding GFP and RFP-NONO monomers (Figure 4E). To then evaluate the docking conformation of the NONO/STAT3 complex, we performed a protein-protein docking simulation using ClusPro 17. The ClusPro scores for the docking model in this instance were -908.5 for the center and -1006.3 for the lowest energy region, suggesting a favorable binding mode (Figure 4F).

NONO was further found to co-localize with STAT3 in the nucleus (Figure 4G), suggesting that these factors interact functionally. Indeed, the NONO protein interacted with the *CCND1* gene promoter region, which is a target of STAT3 (Figure 4H). In further support of the contention that NONO and STAT3 proteins interact at STAT3 target promoter sites, ectopically expressed NONO was observed to directly activate a STAT3 reporter construct, and this activity increased in the presence of STAT3 (Figure 4I). Consistently, the silencing of NONO decreased *STAT3* promoter activity, which was reversed by the re-introduction of STAT3; again suggesting that STAT3 transcriptional activity is regulated by NONO (Figure 4J). We speculated that NONO may mediate part of its activity by stabilizing STAT3, since RBPs frequently affect RNA and protein stability to maintain various cellular functions 36. After treatment with the protein synthesis inhibitor cycloheximide, we measured the protein expression of STAT3 in NONO-suppressed cells. As shown in Figure 4K, STAT3 was more rapidly degraded in NONO-suppressed cells treated with cycloheximide. Blocking transcription by exposing the cells to actinomycin D produced similar results (Figure 4L). Thus, our data suggest that NONO regulates STAT3 expression and function by regulating both RNA levels and protein stability through direct protein-protein interactions, thereby maintaining the oncogenic function of STAT3.

### Clinical relevance of NONO-STAT3 in TNBC

**Figure 5:**
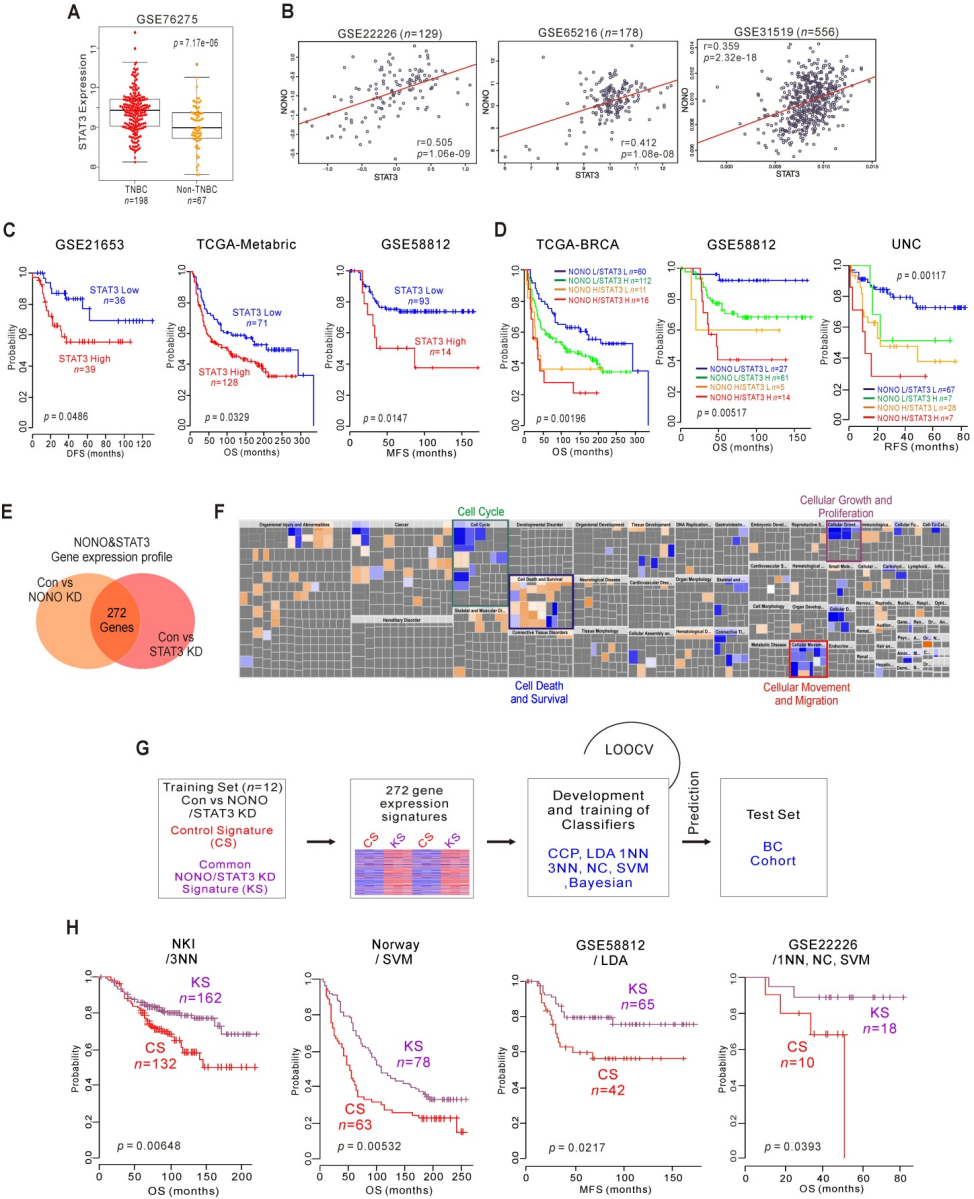
** NONO-STAT3 is clinically associated with TNBC (A)** STAT3 expression in TNBC and non-TNBC patients in the BC cohort. **(B)** Correlation of NONO and STAT3 gene expression in the indicated BC population, including TNBC patient cohorts. Scatter plots of NONO and STAT3 in the TNBC cohorts are shown. **(C-D)** Indicated patient cohorts were divided in two or four groups by the relative expression of NONO and STAT3. A log‐rank test was applied to estimate the significance of the differences. **(E)** Gene expression signature specific to the loss of NONO or STAT3 expression by shRNA or siRNA, respectively in MDA-MB-231 cells. Genes in the Venn diagram were selected by applying a two-sample Student's* t*-test (*P* < 0.001; more than 1.5 fold). The orange and pink circles represent genes whose expression patterns are significantly associated with the loss of NONO or STAT3*,* respectively. **(F)** IPA of the genes differentially expressed after NONO and STAT3 silencing. **(G)** Schematic diagram of prediction model generation and evaluation of predicted outcomes based on a shared gene expression signature of NONO and STAT3 in MDA-MB-231 cells. A shared gene expression signature was used to form a series of classifiers that estimated the probability of how much the expression pattern of BC patients was similar to the shared signature, control signature (CS) vs. knockdown signature (KS). **(H)** Kaplan-Meier plots of OS or MFS breast cancer patients in the indicated cohorts were predicted using the gene expression signature as a classifier. The differences between groups were significant as indicated (log-rank test). LOOCV, Leave-One-Out Cross-Validation; CCP, compound covariate predictor; 1NN, one nearest neighbor; 3NN, three nearest neighbors; NC, nearest centroid; SVM, support vector machines; LDA, linear discriminator analysis.

Since NONO is associated with the clinical outcomes in cancer patients, and STAT3 is regulated by NONO and is involved in its function, we investigated the clinical relevance of STAT3 in samples from BC patients. As expected, STAT3 expression was markedly higher in TNBC compared with non-TNBC tissues (Figure 5A and S7A) and was positively correlated with NONO mRNA expression in various BC patient cohorts (Figure 5B and S7B). Kaplan-Meier analysis of dichotomized STAT3 gene expression revealed that higher expression levels are associated with poorer clinical outcomes in TNBC (Figure 5C and S7C) and that higher NONO and STAT3 levels are associated with significantly poorer patient survival (Figure 5D and S7D).

As transcriptional regulators, NONO and STAT3 influence downstream gene expression to maintain their oncogenic properties. We investigated the gene networks shared between NONO and STAT3 by comparing the gene expression signatures specific to the silencing of both factors 37 (GSE85579) in MDA-MB-231 cells. The resulting Venn diagram in Figure 5E indicates that a substantial number of genes could be identified as downstream targets of both factors, suggesting that the biological activity of NONO might be dependent on STAT3. To better understand the gene network traits that are common to NONO and STAT3, 272 gene signatures were analyzed by IPA. This analysis, shown in Figure 5F, revealed that the common gene signatures between these factors are highly associated with cancer cell growth and proliferation, and with the cell cycle, death, movement, and migration, consistent with the results shown in Figure 3C. We next tested the clinical relevance of these shared signatures by applying a previously established prediction strategy that employs multiple different algorithms 12, 38 (Figure 5G). As expected, the shared gene expression signatures were significantly associated with patient survival and disease recurrence in BC patients when judged using the predicted outcomes of various classifiers 38. Patients with knockdown signatures (KS signatures) showed better prognosis and vice versa (Figure 5H and S8). Taken together, our present findings indicate that NONO is functionally associated with STAT3 and can dictate TNBC clinical outcomes.

### NONO silencing sensitizes TNBC cells to chemotherapeutics

**Figure 6:**
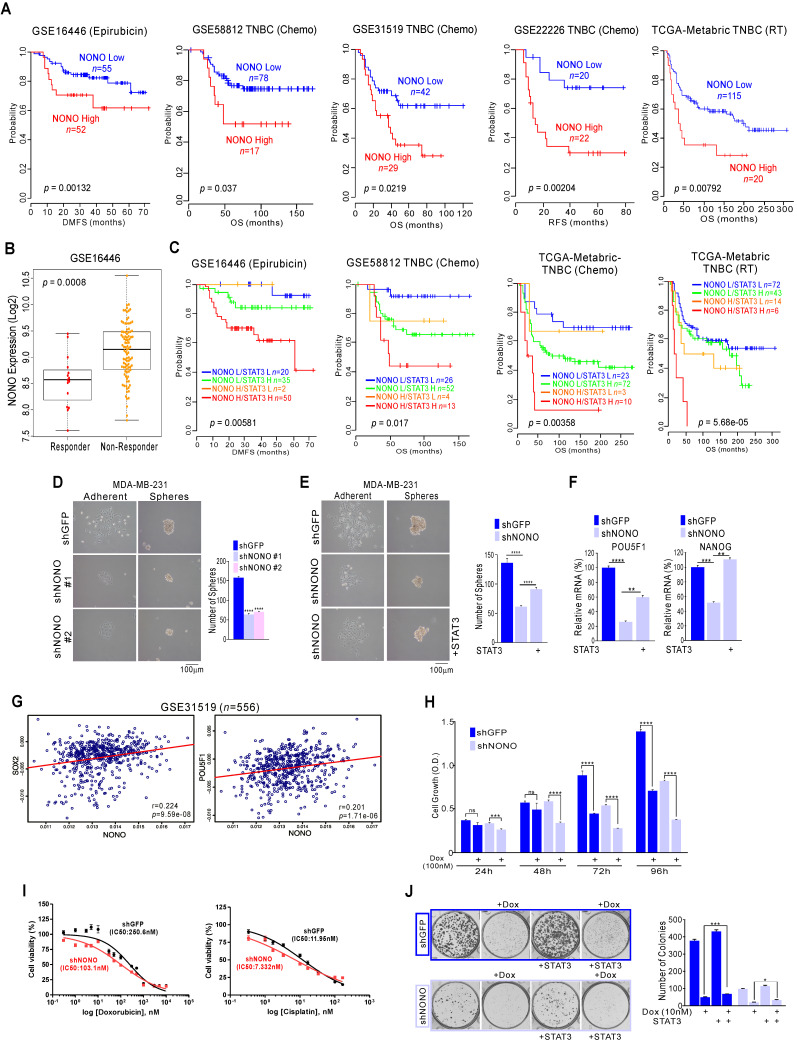
** NONO confers drug-resistance via STAT3 gene regulation in TNBC (A, C)** Kaplan-Meier plots of the OS or distant-metastasis free survival outcomes in patients from the indicated cohorts.** (B)** NONO expression in the indicated patient groups. **(D-F)** Representative images, number of spheres, and mRNA expression levels by qRT-PCR from MDA-MB-231 cells **(F)**, infected with the indicated shRNAs and STAT3 cDNA. **(G)** Correlation of NONO and POU5F1 or NANOG gene expression in the indicated TNBC patient cohorts. Scatter plots of NONO and correlated genes in the TNBC cohorts are shown. **(H-I)** MDA-MB-231 cells were infected with shNONO or shGFP and treated with doxorubicin for the indicated times. The cells were then analyzed for CCK8 assay **(H)** and IC50 calculation** (I)**. **(J)** Clonogenic survival was quantified in a colony formation assay. NONO knockdown cells were treated with doxorubicin and stained cells were counted to verify the re-introduction of STAT3. All results are means plus standard deviations from three independent replicates (*p < 0.05, **p < 0.01, ***p < 0.005, and ****p < 0.001).

The responsiveness to chemotherapy and radiation therapy is a strong indicator of the clinical outcome in TNBC patients 5, 39. Genomic analysis has shown that the high expression of NONO is related to poorer prognosis in TNBC; as shown in Figure 1, our current analysis found NONO highly associated with chemotherapeutic responsiveness. We performed further detailed analysis to investigate whether NONO expression is associated with the treatment response. Epirubicin is an anthracycline agent widely used in TNBC; our genomic datasets indicated that NONO expression was associated with poor prognosis in epirubicin-treated ER-negative patients and other agents-treated patients (Figure 6A). We also observed that NONO expression was significantly higher in treatment non-responders (Figure 6B). Furthermore, high NONO levels were associated with a poorer prognosis in TNBC or basal-like tumor patients who had undergone chemotherapy or radiation treatments (Figure S9A), and both NONO and STAT3 expression was also found to contribute to drug resistance (Figure 6C and S9B). These findings implicate NONO in the drug-responsiveness of TNBC.

Previous reports have suggested that drug-resistant cancer cells show features of cancer stem-like cells (CSCs) and that STAT3 is one of the major factors contributing to CSC proliferation 40. Since NONO is involved in STAT3 regulation and is associated with drug-resistance, we tested whether NONO influences CSC proliferation via STAT3 regulation. The sphere formation assay found CSC proliferation to be significantly decreased in NONO-silenced cancer cells (Figure 6D and S10A); this was reversed by reintroducing STAT3 (Figure 6E) and reduced the expression of CSC markers by NONO-silencing was recovered by reintroducing STAT3 (Figure 6F). Furthermore, NONO correlated with the CSC markers SOX2, NANOG, and POU5F1/Oct4 in TNBC patients (Figure 6G and S10B). These results clearly indicate that NONO regulates CSC proliferation via STAT3 and that NONO promotes drug resistance in TNBC cells by influencing CSC proliferation.

We next tested whether NONO expression is associated with the drug or radiation treatment response using NONO-silenced TNBC cell lines. When NONO expression was silenced, MDA-MB-231 cells became sensitive to the doxorubicin standard therapy used in TNBC patients (Figure 6H), suggesting that NONO accounts for the resistance of TNBC cells to this drug. In addition, other treatments commonly used in TNBC patients, such as cisplatin and radiation, were found to be more effective in NONO-silenced MDA-MB-231 cells (Figure and S11A and S11B) and drug IC50 in NONO silenced cells was lower in parental cell (Figure 6I). In addition, the recovery experiment revealed that resistance to the chemotherapeutic-drugs was dependent on STAT3 expression (Figure 6J and S11C). These results demonstrated that silencing of NONO improves the efficacy of chemotherapeutics and radiation therapy in TNBC models.

### Drug screening for NONO inhibitors in TNBC

**Figure 7:**
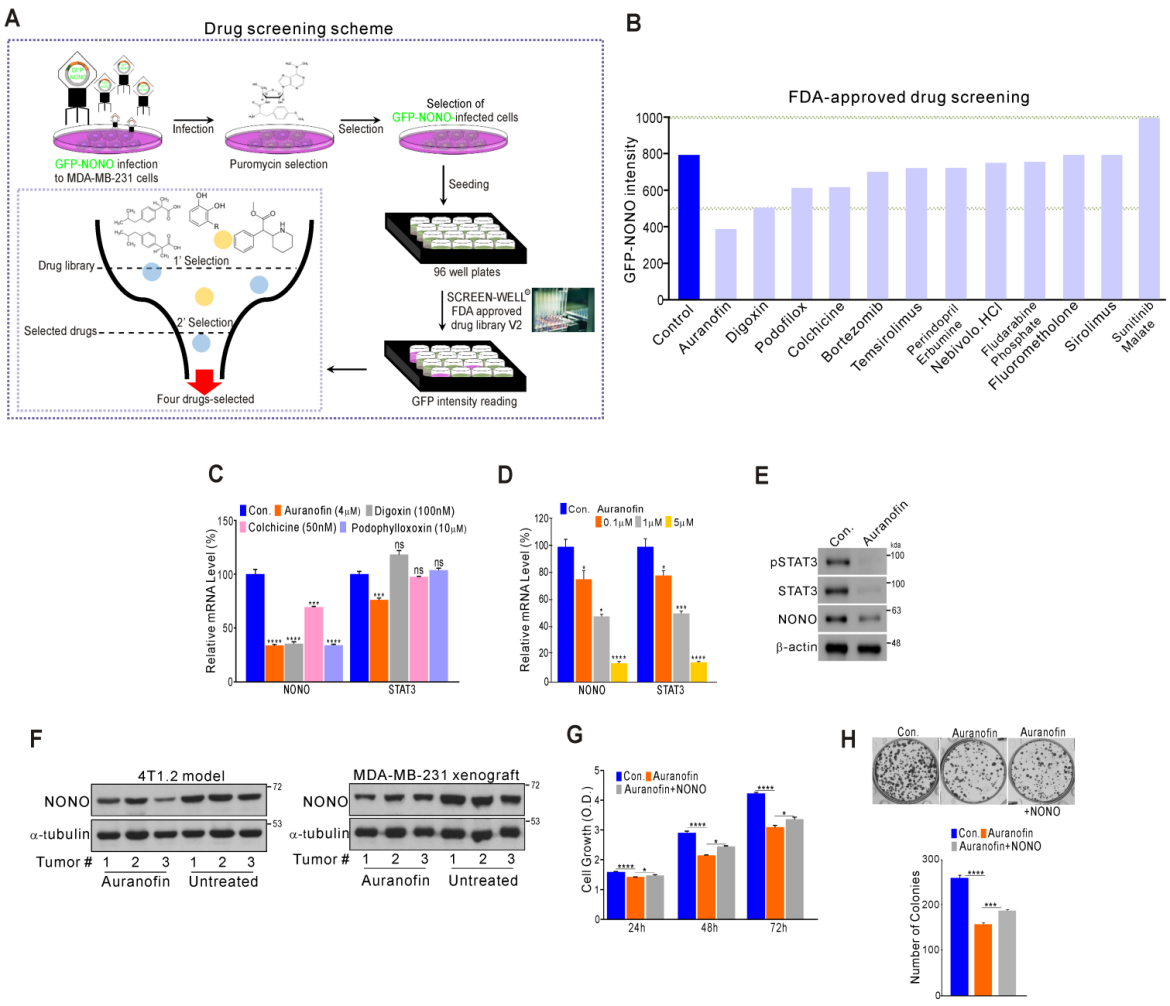
** High-throughput drug screening for NONO inhibitors in TNBC (A)** Overall drug screening scheme. **(B)** Results of drug screening. GFP-NONO fluorescence intensity is indicated relative to control samples. **(C-E)** MDA-MB-231 cells were treated with indicated drugs, and the cells were then analyzed by qRT-PCR with the indicated primers **(C-D)** and WB (E). **(F)** Western blot analysis of the indicated tumor tissues 41. **(G and H)** MDA-MB-231 cells were treated with auranofin, transfected with NONO cDNA, and then analyzed in a CCK8 **(G)** and colony formation assay **(H)**. All results are means plus standard deviations from three independent replicates (*p < 0.05, **p < 0.01, ***p < 0.005, and ****p < 0.001).

Since NONO was found to be critically associated with cancer cell growth and clinical outcomes, we investigated whether the direct targeting of NONO would be an effective way to suppress cell proliferation in TNBC. To identify novel drugs capable of suppressing NONO activity, we performed high-throughput screening of a library of FDA-approved drugs comprising 770 compounds (Screen-Well ® FDA approved drug library V2). These compounds have previously well characterized in terms of safety, biological activity, and function. MDA-MB-231 cells were infected with GFP-NONO cDNA and selected with puromycin. The cells were then seeded and treated with the drug library; then, the GFP-signal intensity was measured (Figure 7A). The first selection results revealed that 12 compounds in the library inhibited the GFP-NONO signal intensity, and a repeat validation indicated that 11 of these compounds suppressed the GFP-NONO signal (Figure 7B). We tested whether four of these compounds (auranofin, digoxin, colchicine, and podophyllotoxin) suppressed NONO and the STAT3 gene expression level downstream of NONO. Auranofin significantly suppressed NONO and both the STAT3 mRNA and protein expression level in MDA-MB-231 cells (Figure 7C-E). The murine 4T1.2 syngenic model reflecting TNBC phenotypes 41 and the xenograft model using MDA-MB-231 revealed that NONO expression was decreased by auranofin treatment (Figure 7F). To next whether the cell growth inhibition by auranofin is dependent on NONO gene expression, we performed a rescue experiment. Cancer cell growth suppression by auranofin was indeed found to be partially reversed by re-introduction of NONO (Figure 7G and H), demonstrating that the growth inhibitory effects of auranofin are mediated by NONO expression targeting.

Our data thus indicate that auranofin might be a candidate NONO inhibitor particularly promising as an anti-cancer therapeutic agent.

## Discussion

A detailed molecular understanding of the properties and functions of oncogenes is vital to uncovering novel molecular targets and developing alternative treatments for different types of cancer. RBPs can drive tumorigenesis in a similar manner to oncogenes by altering cancer initiation, progression, and metastasis 20. Indeed, further dysregulation of RBPs frequently detected in various cancer types.

**Figure 8:**
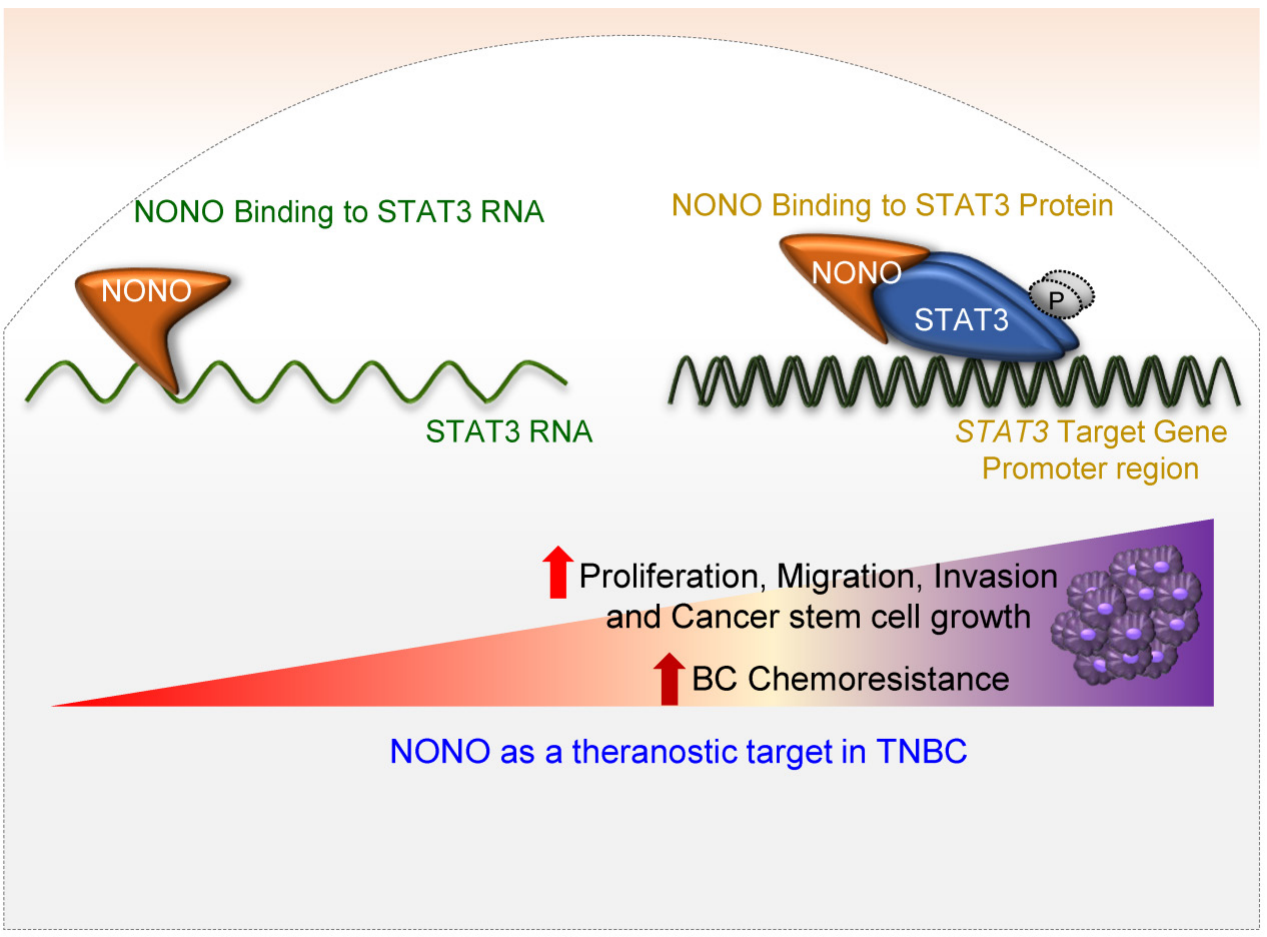
** Schematic representation of the NONO-STAT3 gene regulation mechanism** NONO regulates STAT3 expression via its direct interactions with STAT3 RNA and protein. NONO directly binds to the STAT3 RNA region and recruits the STAT3 protein to STAT3 target promoters, such as the *CCND1* promoter. NONO also modulates STAT3 transcriptional activity. Finally, NONO modulates STAT3 stability and thereby contributes to cancer cell proliferation and chemoresistance in TNBC.

In our present study, we have identified NONO as a pivotal RBP in the modulation of TNBC cell growth (Figure 8). Whereas NONO upstream regulators such as Ets-1 25 and CRTC/LINC00473 42 have already been defined, the NONO downstream targets that drive tumorigenesis have not been previously characterized. We noted that the NONO-STAT3 axis mediates cancer cell growth and drug-sensitivity in TNBC. Importantly, NONO appears to function through its binding to both STAT3 RNA and protein. STAT3 and NONO have been shown to facilitate cancer cell growth, invasion, and migration 31. STAT3 directly regulates various oncogenes such as *COX2* and the *ID1*,* CCND1*,* VEGF*,* MMP,* and *ILs* families as downstream targets to drive carcinogenesis 43 and is hence a promising target for the treatment of several malignant tumors 44. Our present data suggest that the oncogenic potential of NONO is largely dependent on STAT3 expression (Figures 3-6). To exert its typical function, each RBP directly binds to its specific and preferentially recognized RNA sequence and thereby participates in various key cellular events such as transcription, translation, stability, localization, and degradation 20. We found from our present analysis that NONO directly interacts with a putative binding site within the STAT3 RNA region (Figure 4A-C). Surprisingly, in addition to its role in stabilizing STAT3 RNA, NONO directly binds the STAT3 protein and increases its stability and activity. This allows NONO to alter STAT3 function through multiple cooperative mechanisms (Figure 4 and 8).

STAT3 along with NONO confer chemoresistance, which is highly correlated with the DNA repair pathway; previous studies have already revealed that NONO does influence the DNA repair pathway 45-47. Our data partially provide that NONO maybe confer chemoresistance through its DNA repair pathway regulation.

In this study, our systematic analysis using genomic data uncovered various RBPs that are differentially expressed in TNBCs and other BC types (Figures 1 and S2). Although recent genomic analysis of the transcriptome has indicated that RBPs are highly expressed in tumor tissues compared to normal tissues 48, differentially expressed RBPs have not yet been identified in distinct subtypes of cancer. We observed 10 RBPs that are significantly upregulated in TNBC and are highly associated with the chemoresponsiveness of these tumors. Although in our current study we focused on NONO in our current study, further investigations on other RBPs are warranted since there is a very strong possibility that other members of this family also possess significant oncogenic properties. Indeed, recent investigations have demonstrated that various RBPs have functional roles in ER-positive BC. For example, the MSI2 RBP has been reported to participate in the ESR1 gene regulation 9, while RNPC1 RBP has been found to modulate ESR1 stabilization 49. Although some studies have also previously implicated a role for RBPs in TNBC 8, the underlying mechanisms still remained poorly understood and the clinical relevance unclear. However, our present findings clearly demonstrate that NONO expression is significantly higher in TNBC and that this RBP is functionally associated with cell proliferation in this cancer type. Although further validations will be needed to use NONO as a molecular marker, its tentative application as a therapeutic target in TNBC is highly desirable.

STAT3 activation is known to lead to drug resistance in several cancers 50, 51. This suggests that the NONO-STAT3 axis is crucial for drug responsiveness and thereby for the patients' clinical outcome. Silencing of NONO in TNBC cells, which causes downregulation of STAT3 gene expression, increases their sensitivity to chemotherapeutic drugs (Figure 6). Targeting NONO may thus provide an effective approach to block the oncogenic properties of STAT3 and increase TNBC sensitivity to chemotherapy. As we have demonstrated herein, siRNA administration is a feasible approach to suppress NONO and therefore provides a proof-of-concept (Figure 2J); downregulation of NONO by antisense RNA or by proteolysis-targeting chimeras, both of which are being adopted in various clinical settings, is a potential new treatment approach in TNBC. In addition, the disruption RBP-RNA interactions with small-molecule inhibitors or oligonucleotides has been achieved already in proof-of-concept experiments 52. The crystal structure of NONO has revealed that it forms a heterodimer with SFPQ 53. However, whether this heterodimerization is functionally involved in cancer development and in the assembly of the NONO-STAT3-SFPQ-triple complex is still unclear however; it is therefore yet unknown whether disruption of either this heterodimer or complex will suppress NONO functions.

Based on our initial aforementioned observations, we explored the possibility of using a direct NONO inhibitor in TNBC. Unexpectedly, our drug screening results revealed that auranofin is one of several potential inhibitors of NONO in TNBC (Figure 7). Auranofin is a well-known anti-inflammatory drug used in rheumatoid arthritis 54. Recently, several studies based on a drug-repositioning concept have suggested that auranofin possesses anti-proliferative effects in cancer cells 41. Although our current results hint to auranofin being able to modulate NONO activity, other and more specific targeting agents will also need to be explored.

In conclusion, we report a novel function for the RNA-binding protein NONO in TNBC and reveal a tight relationship between NONO and STAT3. Our findings have increased our understanding of the mechanism underlying the regulation of cancer cell proliferation by specific RBPs and have helped us elucidate novel therapeutic targets for treating TNBC.

## Supplementary Material

Supplementary figures.

Supplementary table.

## Abbreviations

ab: antibody
BC: breast cancer
CH-NP: chitosan-nanoparticle
CSC: cancer stem-like cell
ER: estrogen receptor
FBS: fetal bovine serum
IP: immunoprecipitation
IPA: ingenuity pathway analysis
NONO: non-POU domain containing octamer binding
qRT-PCR: quantitative real-time reverse transcription polymerase chain reaction
RBP: RNA-binding protein
ROC: receiver operating characteristic
shRNA: short hairpin RNA
STAT3: signal transducer and activator of transcription 3
TCGA-BRCA: The Cancer Genome Atlas Breast Invasive Carcinoma
TF: transcription factor
TMA: tissue microarray
TNBC: triple-negative breast cancer
WB: western blotting
