# Supplementary material for: RNA-binding protein NONO contributes to cancer cell growth and confers drug resistance as a theranostic target in TNBC
Source: Theranostics. 2020 Jul 2;10(18):7974–92. doi: 10.7150/thno.45037 (PMC7381744; doi:10.7150/thno.45037)
Supplement: Supplementary file 1 — Supplementary figures. [file thnov10p7974s1.pdf]

Figure S1

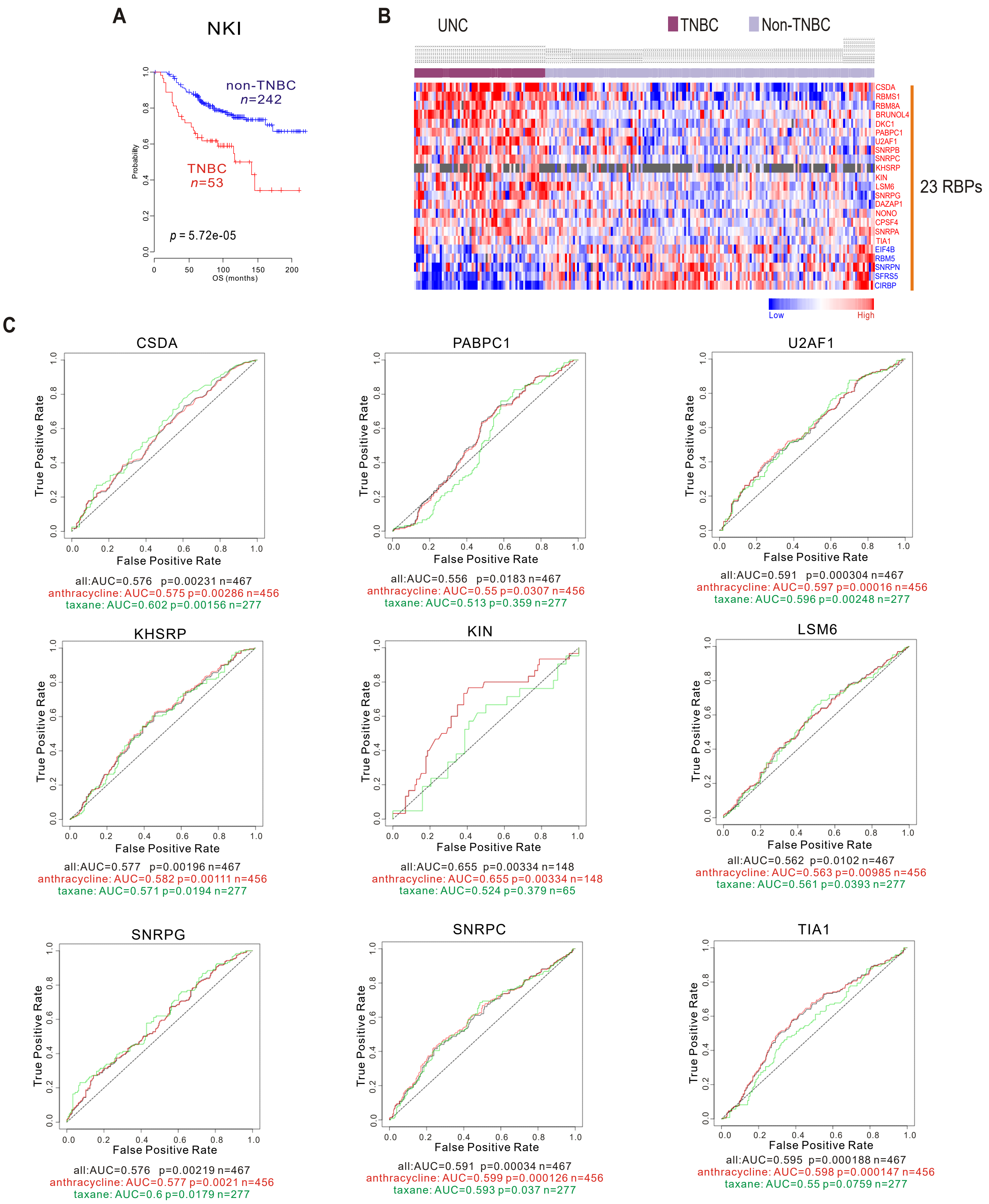

Figure S1 Genomic analysis of RBPs in human breast cancer.

A, Kaplan-Meier(KM) plots of NKI breast cancer patients by BC subtypes. B, clustering of RBPs depending on BC subtypes, TNBC and non-TNBC. C, ROC analysis of indicated RBPs from BC patients.

Figure S2

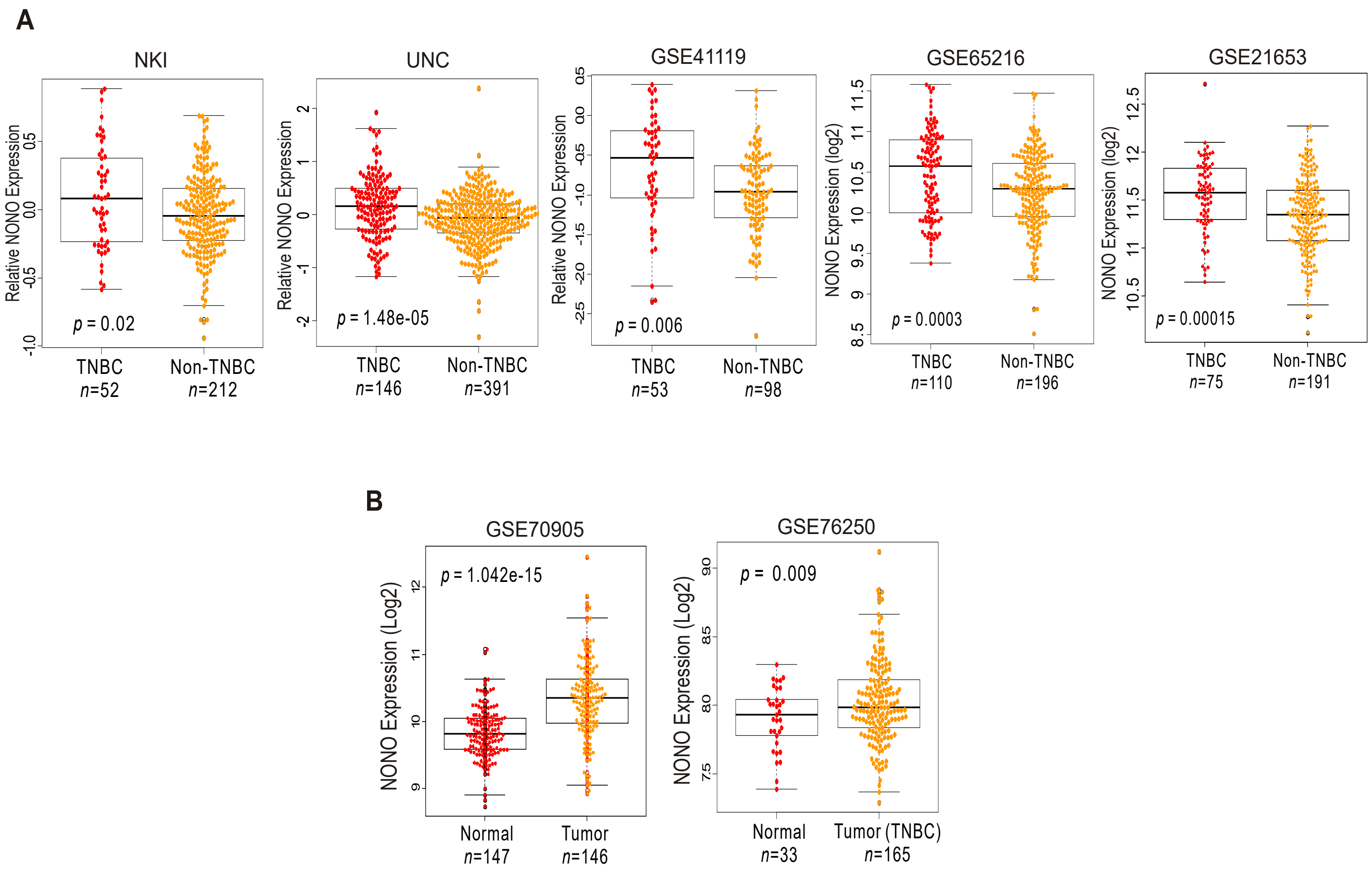

Figure S2 NONO expression in human breast cancer.

A, NONO expression level from indicated BC patient cohorts. B, NONO expression between normal and tumor from indicated BC patients. *p*-value indicates significance of analysis.

Figure S3

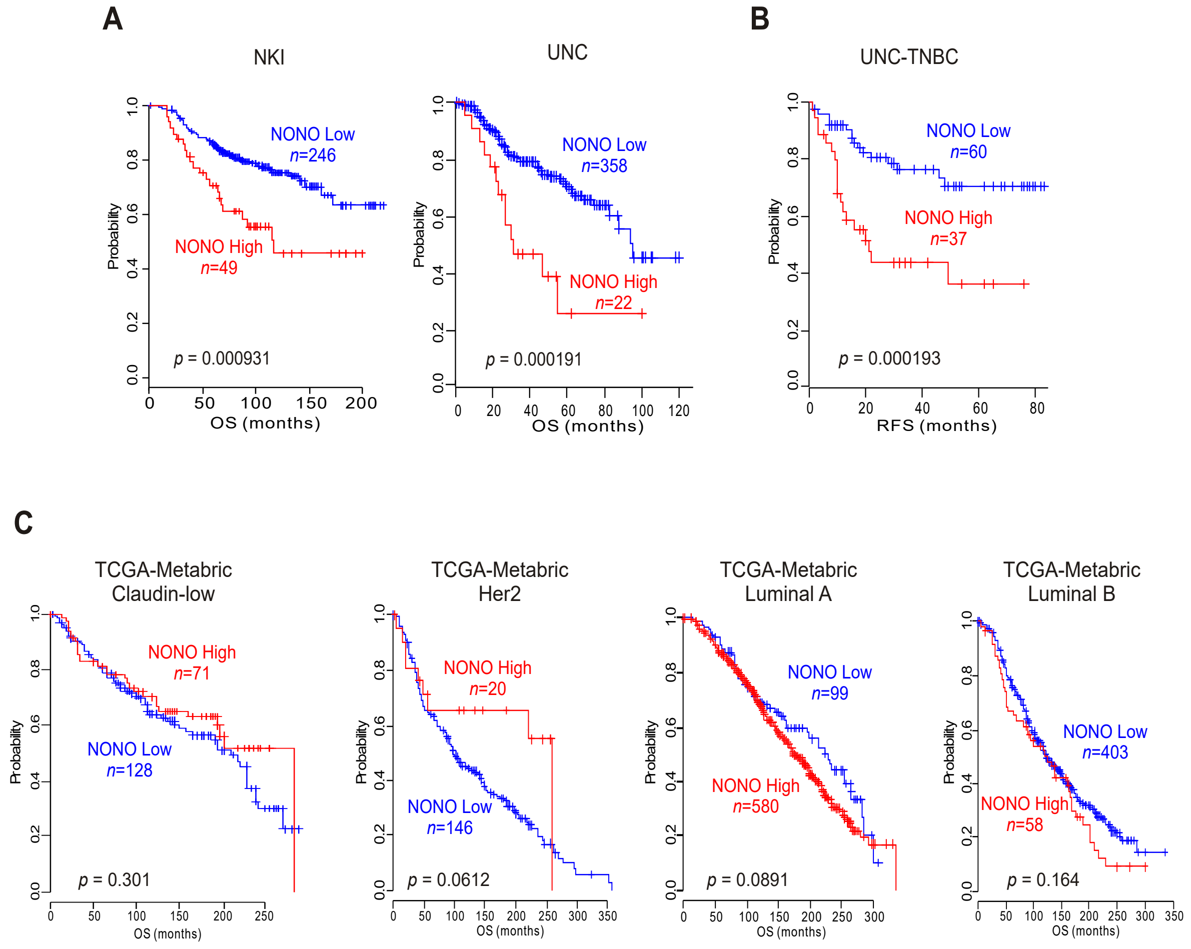

D

TCGA-Metabric Multivariate

Variables	*p* value	HR
NONO	3.9e-04	1.28±0.13
ER Status	0.47	1.06±0.16
Her2	0.0023	1.33±0.18
Lymph node	6.6e-07	1.63±0.12
Grade	0.0026	1.17±0.1

NKI-Multivariate

Variables	*p* value	HR
NONO	0.0108	1.72±0.42
ER	0.52	0.86±0.47
Lymph node	0.21	1.19±0.27
Grade	2.0e-05	1.91±0.30

Figure S3 Survival analysis of NONO in human breast cancer

A, B and C, using indicated cohort, patients were classified according to the expression level of NONO. Kaplan-Meier plots and the log-rank test were used to estimate patient prognosis. D, multivariate analysis with indicated cohorts (HR; hazard ratio).*p*-value indicates significance of analysis.

Figure S4

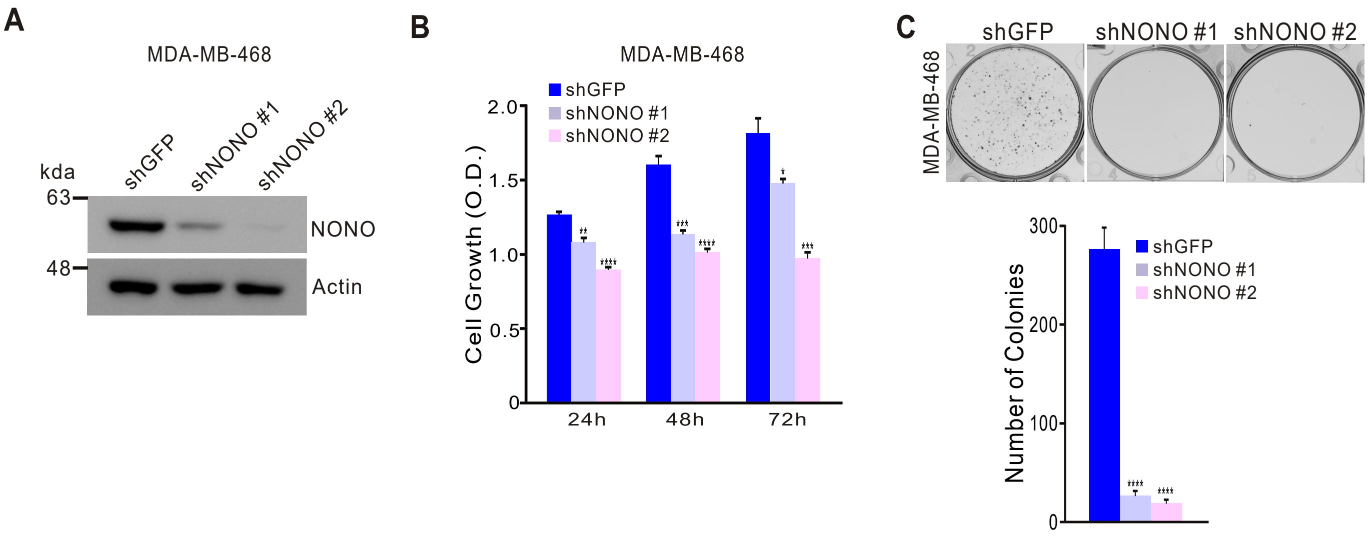

Figure S4 NONO governs cell proliferation in breast cancer cells

A-C, MDA-MB-468 breast cancer cells were stably transfected with shNONO or shGFP. The cells were used for western blot (A), CCK8 (B), and colony formation assay (C). Student t-test (two-tailed) was applied to estimate the significance of cell proliferation (n=3). * indicates significance of cell proliferation (* p<0.05, ** p<0.01, *** p<0.005, and **** p<0.001).

Figure S5

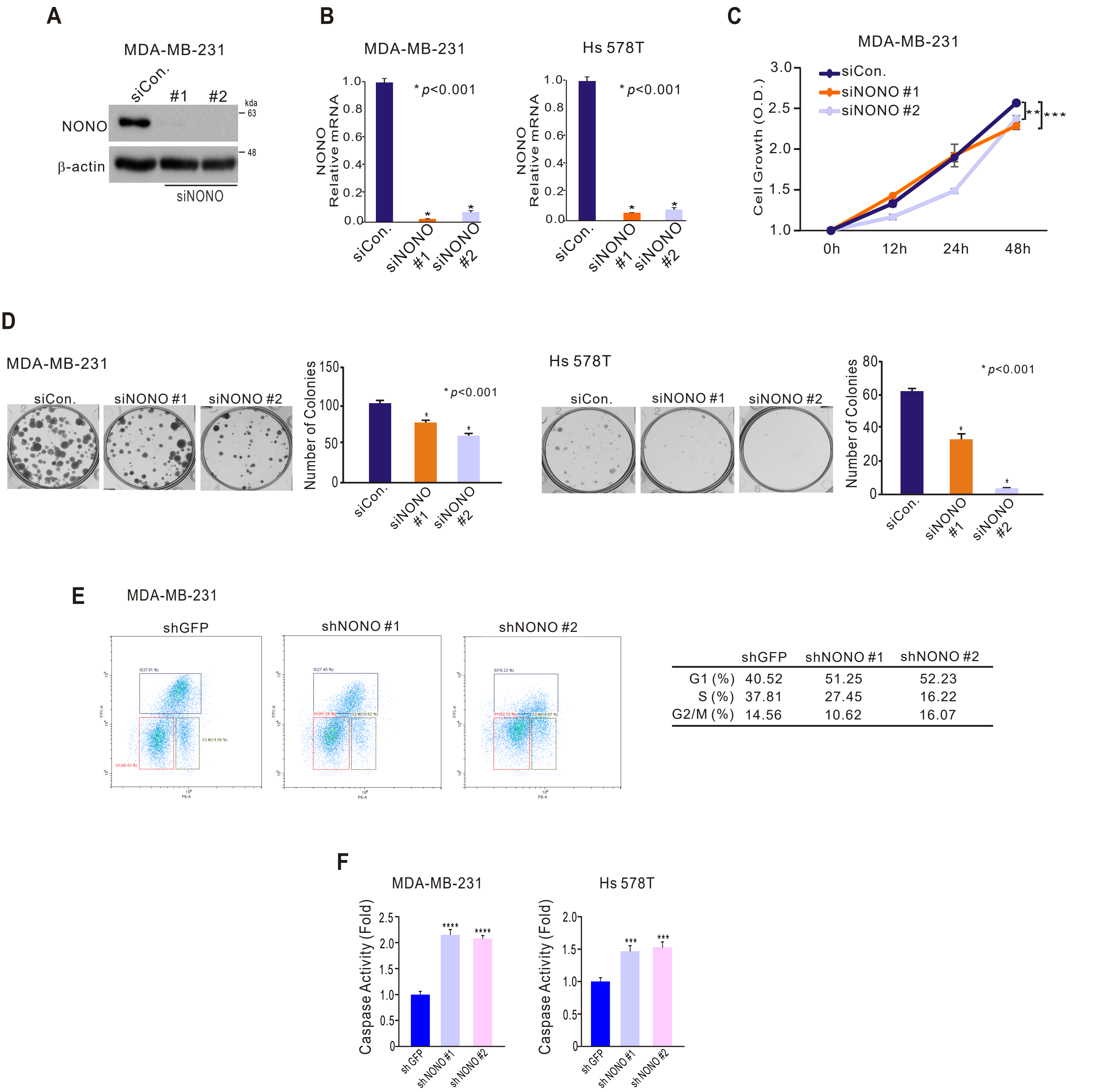

Figure S5 NONO modulates cell growth and proliferation in breast cancer cells

A ~ D, Indicated breast cells were transfected with siNONO or siCon. The cells were used for western blot analysis with indicated antibodies (A), and qRT-PCR (B), CCK8 proliferation assay (C) and colony formation assay (D) (*n*=3). (E-F) MDA-MB-231 cells were infected with shNONO or shGFP and stained with EdU and cell cycle was analyzed by FACS (E) and caspase activity was measured (F). Student t-test (two-tailed) was applied to estimate the significance of assay (* $p<0.05$, ** $p<0.01$, *** $p<0.005$, and **** $p<0.001$).

Figure S6

NONO -binding Rank	Motif	*STAT3* Locus
1	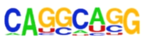	Chr17: 42,313,859 ~ 42,313,866 (M1-1)<br>Chr17: 42,323,456 ~ 42,323,463 (M1-2)
2	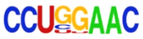	Chr17: 42,367,511 ~ 42,367,518 (M2)
3	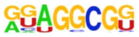	Chr17: 42,344,645 ~ 42,344,652 (M3)
4	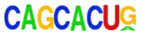	Chr17: 42,345,015 ~ 42,345,022 (M4)

Figure S6 NONO binding motifs on the *STAT3* RNA

Enriched RNA motifs among the defined NONO-binding sequences on the STAT3 locus-based on the RNA immunoprecipitation-sequencing (Ref: Benegiamo G et al., Cell Metabolism 27, 404-418, Feb 6 2018).

Figure S7

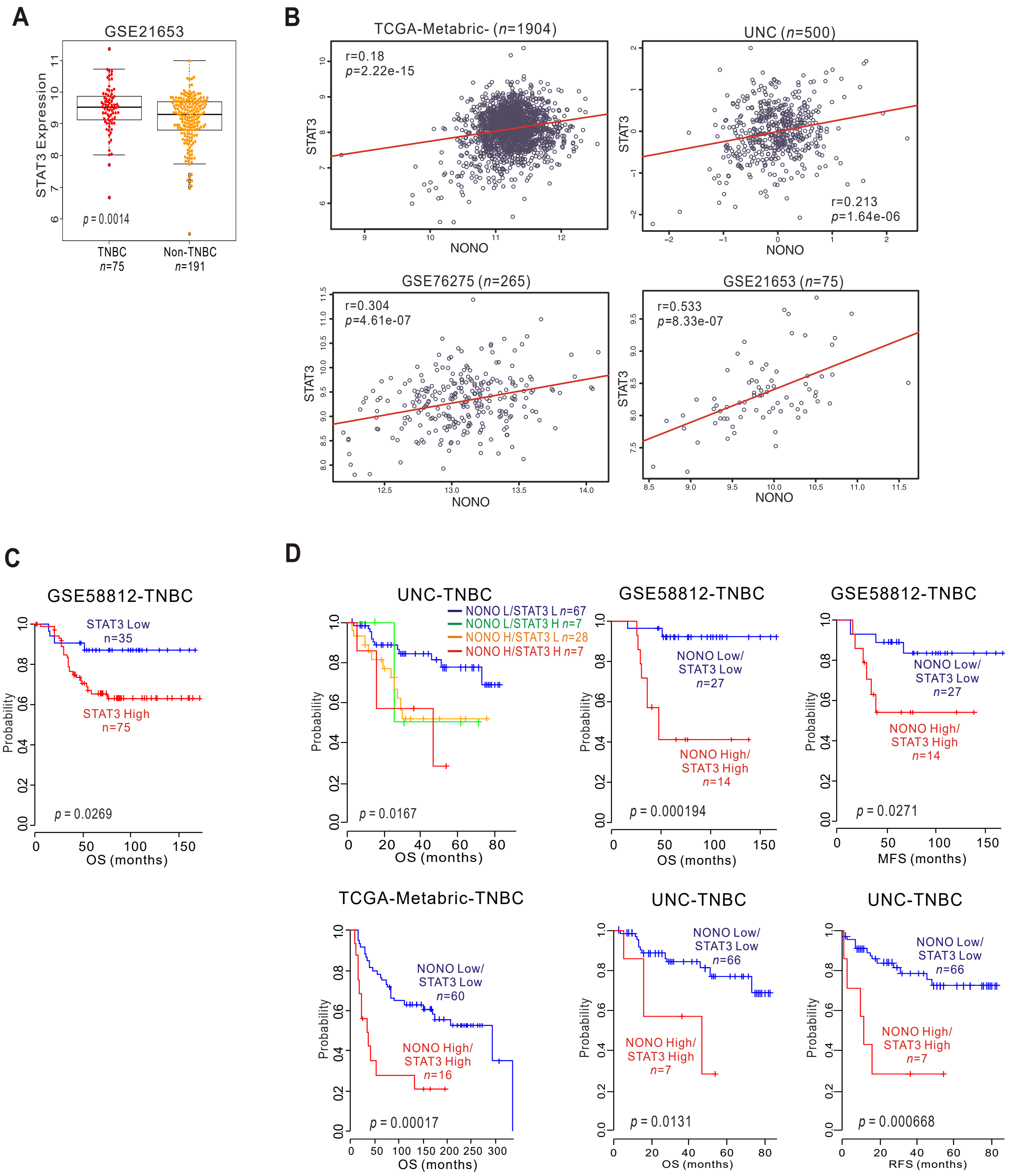

Figure S7 Clinical relevance of NONO and STAT3 in BC

A, STAT3 expression between TNBC and non-TNBC from GSE21653 BC patient cohorts. B, Correlation scatter plots between NONO and STAT3 in indicated BC cohorts (TNBC cohorts:GSE76275, GSE21653). C and D, using indicated cohorts, patients were classified according to the expression level of NONO and STAT3. Kaplan-Meier plots and the log-rank test were used to estimate patient prognosis. *p*-value indicates significance of analysis.

Figure S8

A

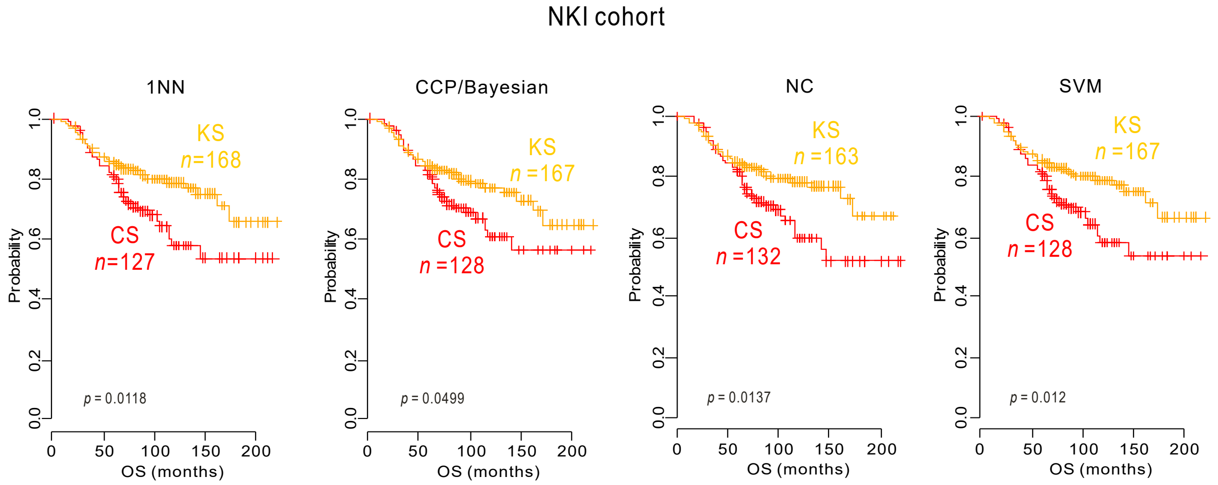

B

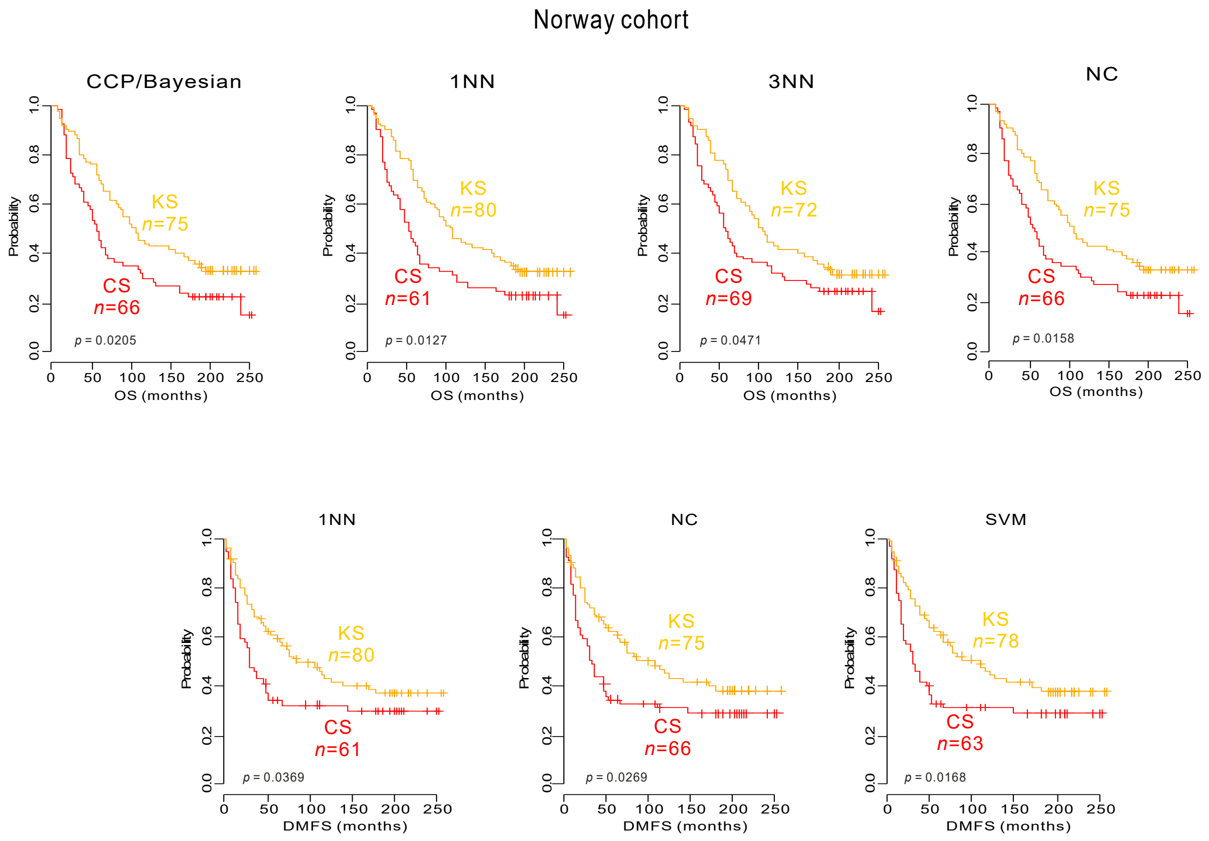

Figure S8 Survival prediction with NONO and STAT3 gene signatures in BC

A and B, Kaplan-Meier plots of OS and DMFS (Distant-metastasis free survival) of BC patients from NKI and Norway cohorts predicted by using shared gene expression signature between NONO and STAT3 with indicated classifiers (CS:Cotrol Signature; KS: Knock down Signature; SC:Compound covariate predictor (CCP), one nearest neighbor (1NN), three nearest neighbor (3NN), nearest centroid (NC), support vector machines (SVM) and linear discriminator analysis (LDA)). *p*-value indicates significance between groups-based on log-rank test.

Figure S9

A

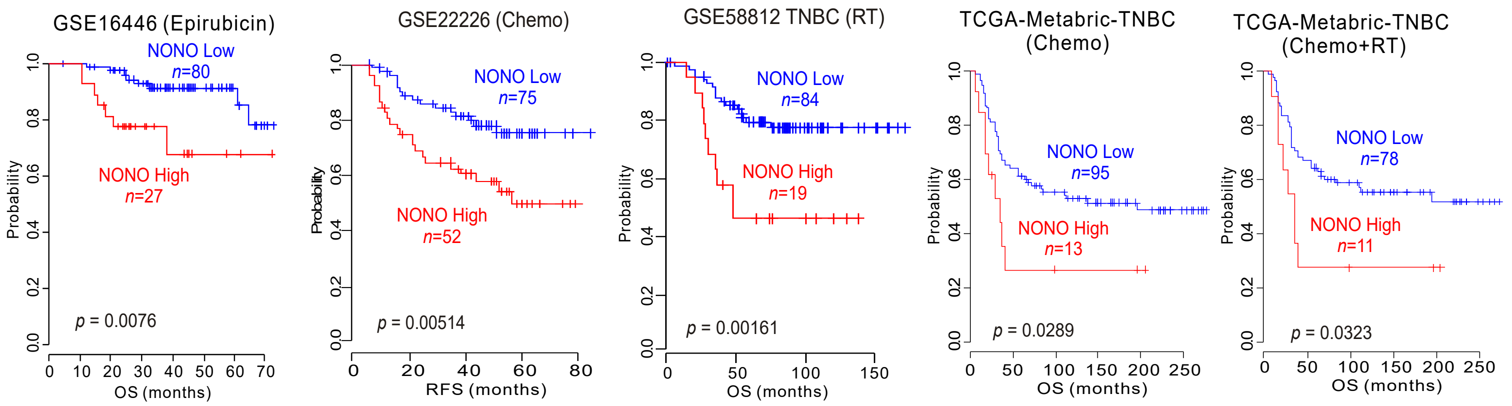

B

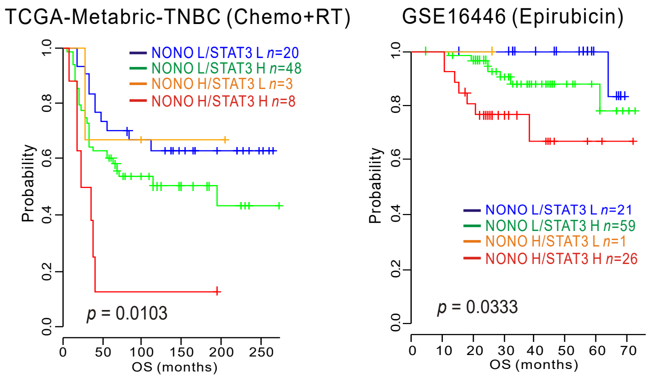

Figure S9 Survival analysis of BC patients by NONO expression

A and B, Kaplan-Meier plots of OS or RFS of BC patients who received indicated theraphy in GSE16446, GSE22226,GSE58812 and TCGA data-based on NONO and STAT3 gene expression. *p*-value indicates significance of analysis.

Figure S10

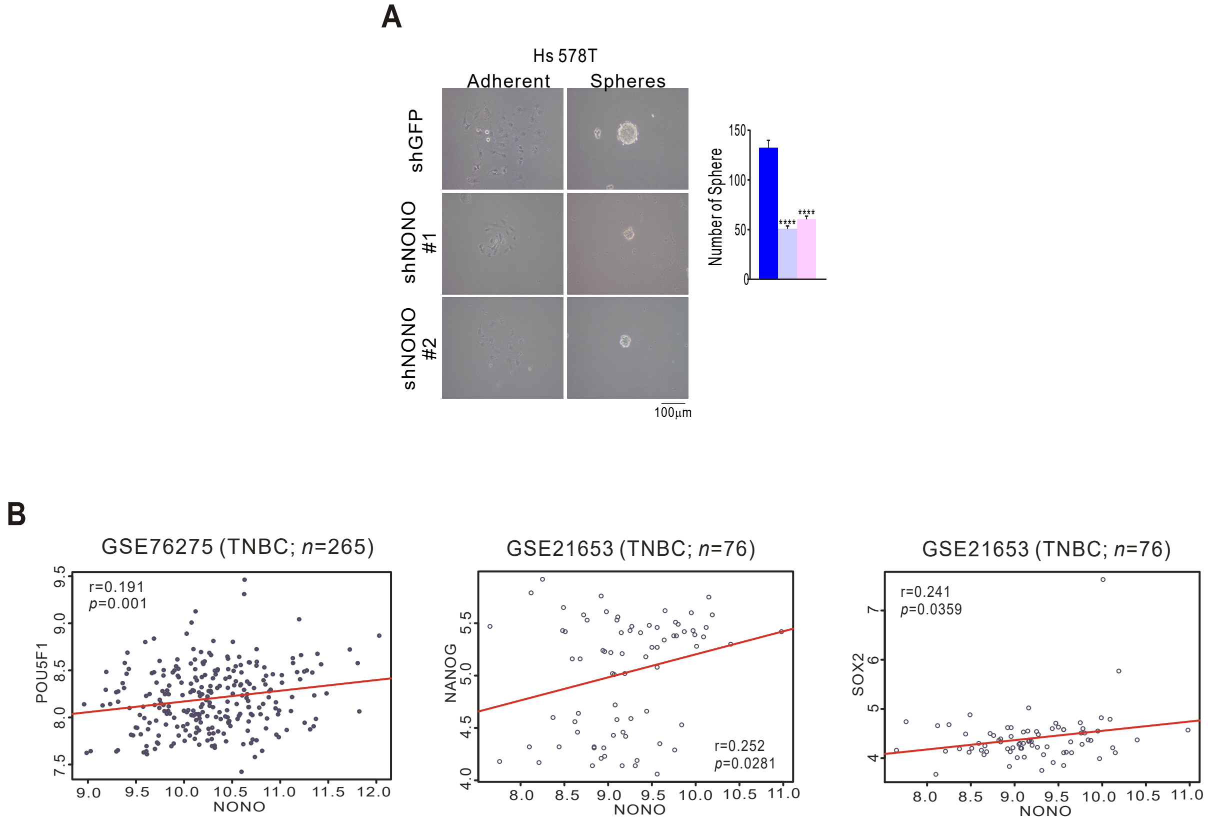

Figure S10 Correlation of NONO and cancer stem cell (CSC) marker in BC

A, Hs 578T cell lines were infected with shNONO or shGFP and sphere formation was measured.
B, Correlation scatter plots between NONO and CSC markers in indicated BC including TNBC cohorts. *p*-value indicates significance of analysis (* $p<0.05$, ** $p<0.01$, *** $p<0.005$, and **** $p<0.001$).

Figure S11

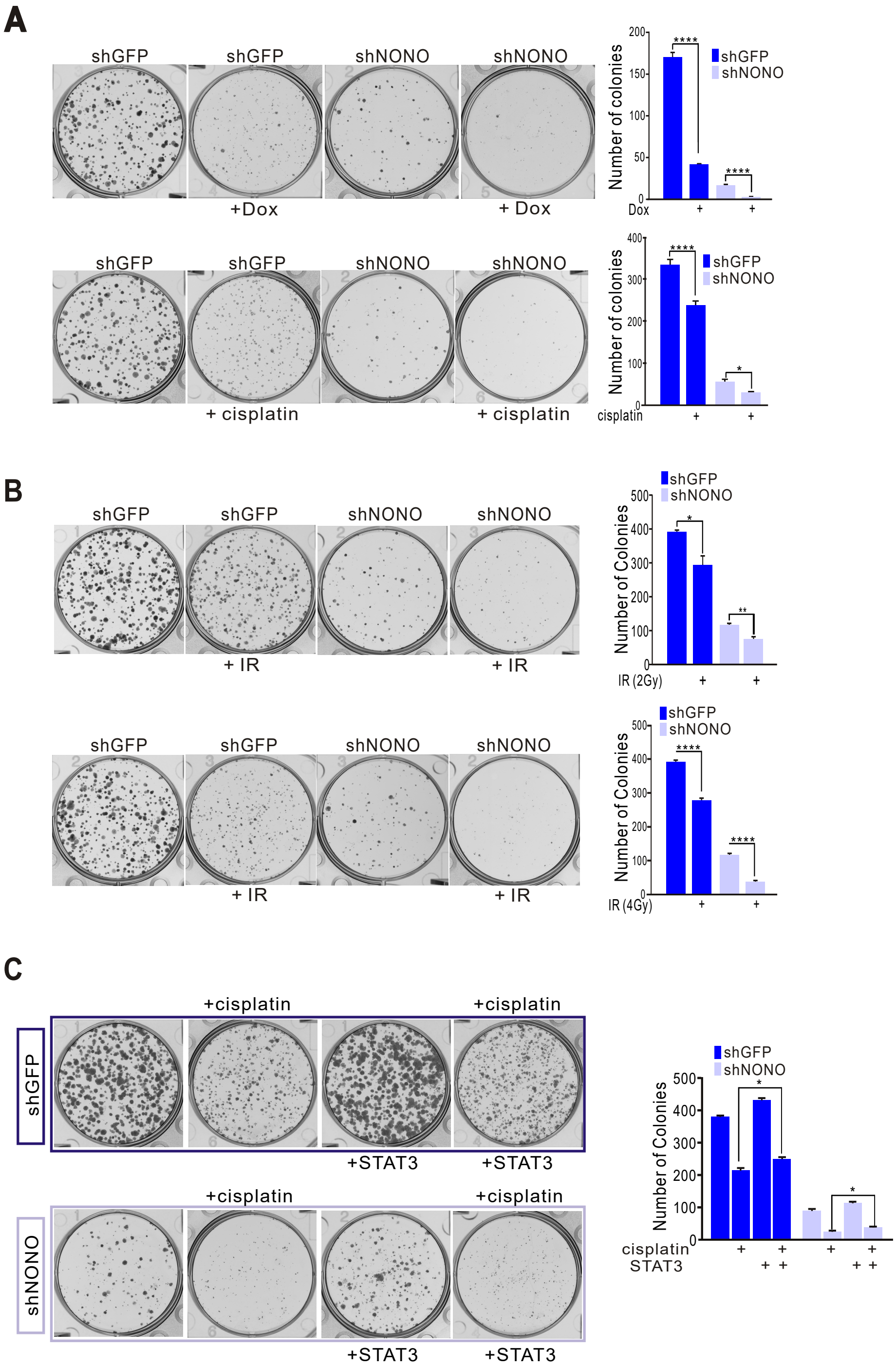

Figure S11 Silencing NONO contributes to chemo and radiation sensitization in breast cancer cells

A-C, MDA-MB-231 breast cancer cells were stably transfected with shNONO or shGFP. The cells were treated with indicated drugs (A; C, Dox:10nM, cisplatin: 3.34nM) or were exposed to radiation (IR; 2 or 4 Gy) (B) and after 14 days, the cells were used for colony formation assay and quantified (n=3). Student t-test (two-tailed) was applied to estimate the significance of cell proliferation. * indicates significance of cell proliferation, * p<0.05, ** p<0.01, ***p<0.005, and **** p<0.001.
